# Challenges related to data protection in clinical research before and during the COVID-19 pandemic: An exploratory study

**DOI:** 10.3389/fmed.2022.995689

**Published:** 2022-10-10

**Authors:** Teodora Lalova-Spinks, Evelien De Sutter, Peggy Valcke, Els Kindt, Stephane Lejeune, Anastassia Negrouk, Griet Verhenneman, Jean-Jacques Derèze, Ruth Storme, Pascal Borry, Janos Meszaros, Isabelle Huys

**Affiliations:** ^1^Clinical Pharmacology and Pharmacotherapy, Department of Pharmaceutical and Pharmacological Sciences, KU Leuven, Leuven, Belgium; ^2^Center for IT & IP law (CiTiP), KU Leuven, Leuven, Belgium; ^3^European Organization for Research and Treatment of Cancer (EORTC), Brussels, Belgium; ^4^MyData-TRUST, Mons, Belgium; ^5^University Hospitals Leuven, Leuven, Belgium; ^6^Clinical Trial Center, University Hospitals Leuven, Leuven, Belgium; ^7^Ethics Committee Research, University Hospitals Leuven, Leuven, Belgium; ^8^Center for Biomedical Ethics and Law, Department of Public Health and Primary Care, KU Leuven, Leuven, Belgium

**Keywords:** clinical research, COVID-19, pandemic, life sciences, data protection, legal framework for clinical trials, GDPR

## Abstract

**Background:**

The COVID-19 pandemic brought global disruption to health, society and economy, including to the conduct of clinical research. In the European Union (EU), the legal and ethical framework for research is complex and divergent. Many challenges exist in relation to the interplay of the various applicable rules, particularly with respect to compliance with the General Data Protection Regulation (GDPR). This study aimed to gain insights into the experience of key clinical research stakeholders [investigators, ethics committees (ECs), and data protection officers (DPOs)/legal experts working with clinical research sponsors] across the EU and the UK on the main challenges related to data protection in clinical research before and during the pandemic.

**Materials and methods:**

The study consisted of an online survey and follow-up semi-structured interviews. Data collection occurred between April and December 2021. Survey data was analyzed descriptively, and the interviews underwent a framework analysis.

**Results and conclusion:**

In total, 191 respondents filled in the survey, of whom fourteen participated in the follow-up interviews. Out of the targeted 28 countries (EU and UK), 25 were represented in the survey. The majority of stakeholders were based in Western Europe. This study empirically elucidated numerous key legal and ethical issues related to GDPR compliance in the context of (cross-border) clinical research. It showed that the lack of legal harmonization remains the biggest challenge in the field, and that it is present not only at the level of the interplay of key EU legislative acts and national implementation of the GDPR, but also when it comes to interpretation at local, regional and institutional levels. Moreover, the role of ECs in data protection was further explored and possible ways forward for its normative delineation were discussed. According to the participants, the pandemic did not bring additional legal challenges. Although practical challenges (for instance, mainly related to the provision of information to patients) were high due to the globally enacted crisis measures, the key problematic issues on (cross-border) health research, interpretations of the legal texts and compliance strategies remained largely the same.

## Introduction

The COVID-19 pandemic brought global disruption to health, society, and economy, including the conduct of clinical research (1, 2). In the European Union (EU), the legal and ethical framework for health research is complex and highly divergent (1, 3). Many challenges exist in relation to the interplay of the various applicable rules, and, in particular, with respect to the compliance with the EU General Data Protection Regulation (GDPR) (3–10). As evidenced by a report prepared for the European Commission (hereafter: the Commission), variations in the national level application of the GDPR have led to fragmentation making cross-border cooperation for research difficult (4). Moreover, various sources suggest that, in many EU Member States, GDPR compliance in the scope of health research is under the scrutiny of ethics committees, even though the data protection rules do not prescribe a specific role for them (11, 12).

During the pandemic, clinical research stakeholders were faced with additional multi-faceted challenges linked to the management of studies (e.g., sufficiently protecting the patients’ safety and integrity, addressing the continuity of studies during the disruption caused by lockdowns and other containment measures, and maintaining efficient communication with authorities and study subjects) (1). Several timely guidelines were issued during the first COVID-19 waves (13, 14). It was repeatedly affirmed by the European Data Protection Board (EDPB) and the European Data Protection Supervisor (EDPS) that the GDPR does not hinder measures taken in the fight against the pandemic. However, the European Medicines Agency (EMA) and national regulatory bodies’ emergency guidance did not address the GDPR application in the context of clinical research in depth, with the exception of comments related to remote source data verification (SDV) and obtaining informed consent (1).

Currently, the Commission is actively working on several policy initiatives that have the potential to add new opportunities, but also to further complexify the legal framework for health research. These include the recently adopted Data Governance Act (DGA), the proposal for a Data Act (DA), and the proposal for a Regulation on the European Health Data Space (EHDS) (15–17). All acts are part of a set of measures comprising the European Strategy for Data (18). The central aim is to facilitate access to and (re-)use of data, including for the purpose of scientific research, while preserving and further strengthening individuals’ control over their personal data. At present, there is not enough clarity as to how these new acts will fit within the existing legal framework for research in general, and what their impact would be on the conduct of clinical research, in particular. However, concerns about some of the new mechanisms that are to be established with these legislative acts (such as data altruism) are already being voiced (19).

The EDPB and the EDPS affirmed that the GDPR provides for the processing of personal data for the purpose of scientific research during the pandemic (13, 14, 20). However, it has not yet been investigated how compliance with the GDPR in the context of clinical research was affected during the pandemic and whether the COVID-19 crisis necessitated significant changes in company/academia compliance strategies. This gap in scholarship constitutes part of a broader lack of empirical research about the implementation and impact of the GDPR and its national derogations (8), that has only recently started to be filled by studies such as that of McLennan et al. on the practices and attitudes of Bavarian stakeholders regarding the secondary use of health data during the COVID-19 pandemic (21). Therefore, at times of significant social and legal transformations, we deemed it of high importance to gather evidence about the experience of key stakeholders involved in clinical research on the main challenges and related possible solutions concerning the compliance with data protection rules before and during the COVID-19 pandemic. The results could point to the topics on which training efforts are most necessary. Finally, the perspectives raised by key stakeholders, as critically discussed in this manuscript, may be used to support the planning and design of various cross-border collaborations and consortia in the field of health research.

## Materials and methods

### Study design

A mixed-methods approach was adopted in the form of an embedded design (22). The study was guided by a traditional quantitative methodology (an online survey), while supplemental qualitative data were collected via follow-up semi-structured interviews. The results from the quantitative and qualitative arms of the study were collected and analyzed separately and compared during the interpretation. The survey and interview questionnaires were designed based on a scoping review of the relevant literature and the research aims. The scoping literature review identified that more research about the impact of the pandemic on the application of data protection rules was required. In addition, representatives of the three targeted stakeholder groups were involved in the design of the questionnaires as co-authors.

The survey was made available in English via the Qualtrics platform. It consisted of two main parts: (1) compliance with the GDPR for clinical studies, and (2) general aspects related to obtaining (electronic) IC. The background and results of the second part are described elsewhere.^1^ The topics included in part 1 of the survey were: (i) primary use of personal data for clinical research purposes, (ii) secondary use of personal data for clinical research, (iii) providing GDPR-related information to study participants, (iv) communication with ethics committees, and (v) challenges related to compliance with the GDPR prior to and during the pandemic (Supplementary material 1). The survey included multiple choice questions, Likert scale questions, open-ended questions, and one ranking question. Six experts with backgrounds in medicine, law, clinical research, and pharmaceutical sciences were involved in the pilot testing, and the think-aloud technique was used (23).

Follow-up semi-structured interviews were performed in English via Microsoft Teams and Zoom. The interview guide (Supplementary material 2) was focused on the same topics as the survey questionnaire.

For the purposes of the survey and interview questionnaires, “primary (prospective) use” was defined as the use of data directly collected and used for the purposes of clinical studies. For example, primary use would be use in projects that were named in the study protocol at the time of data collection (24). Pursuant to the GDPR, the processing of personal data must be based on a valid legal basis [Article 6(1)], Table 1A. One possible legal basis is consent [Article 6(1)(a)], which should be distinguished from informed consent to participate in clinical trials, as foreseen under the Clinical Trials Regulation. In addition, if special categories of data are processed (such as data concerning health – e.g., medical history, examination of results), a special condition under Article 9(2) must be identified to justify an exception to the prohibition of processing such data under Article 9(1), Table 1B. Explicit consent, the protection of vital interests of the data subject, public interest reasons in the area of public health, purposes of preventive or occupational medicine, or scientific research purposes, are examples of such special conditions. As discussed in EDPB Opinion 3/2019, there are two main categories of processing operations in a clinical trial, namely: (1) processing operations purely related to research purposes and (2) processing operations related to safety purposes (e.g., notification of adverse events to competent authorities) (24).

**TABLE 1:** Overview of **(A)** legal bases under Article 6(1) GDPR and **(B)** special conditions relevant for clinical research under Article 9(2) GDPR.

(A) Article 6(1) GDPR	
Consent	*(a) “the data subject has given consent to the processing of his or her personal data for one or more specific purposes”*
Contract	*(b) “processing is necessary for the performance of a contract to which the data subject is party or in order to take steps at the request of the data subject prior to entering into a contract”*
Legal obligation	*(c) “processing is necessary for compliance with a legal obligation to which the controller is subject”*
Vital interests	*(d) “processing is necessary in order to protect the vital interests of the data subject or of another natural person”*
Public interest	*(e) “processing is necessary for the performance of a task carried out in the public interest or in the exercise of official authority vested in the controller”*
Legitimate interests	*(f) “processing is necessary for the purposes of the legitimate interests pursued by the controller or by a third party, except where such interests are overridden by the interests or fundamental rights and freedoms of the data subject which require protection of personal data, in particular where the data subject is a child”*
(B) Article 9(2) GDPR	
Explicit consent	*(a) “the data subject has given explicit consent to the processing of those personal data for one or more specified purposes (*…*)”*
Vital interests	*(c) “processing is necessary to protect the vital interests of the data subject or of another natural person where the data subject is physically or legally incapable of giving consent”*
Personal data manifestly made public	*(e) “processing relates to personal data which are manifestly made public by the data subject”*
Substantial public interest	*(g) “processing is necessary for reasons of substantial public interest, on the basis of Union or Member State law which shall be proportionate to the aim pursued, respect the essence of the right to data protection and provide for suitable and specific measures to safeguard the fundamental rights and the interests of the data subject”*
Preventive or occupational medicine	*(h) “processing is necessary for the purposes of preventive or occupational medicine (*…*)”*
Public interest in the area of public health	*(i) “processing is necessary for reasons of public interest in the area of public health, such as protecting against serious cross-border threats to health or ensuring high standards of quality and safety of health care and of medicinal products or medical devices, on the basis of Union or Member State law which provides for suitable and specific measures to safeguard the rights and freedoms of the data subject, in particular professional secrecy”*
Scientific research	*(j) “processing is necessary for archiving purposes in the public interest, scientific or historical research purposes or statistical purposes in accordance with Article 89(1) based on Union or Member State law which shall be proportionate to the aim pursued, respect the essence of the right to data protection and provide for suitable and specific measures to safeguard the fundamental rights and the interests of the data subject”*

For the purposes of the survey and interview questionnaires, “secondary (retrospective) use” was defined as the re-use (further processing) of personal data that was initially collected and used for another purpose. For example, when health data which was originally collected in the scope of care or in a previous clinical study, is used for the conduct of another study. It was explained to participants that personal data cannot be re-used if that would be incompatible with the initial purposes for its collection and use.^2^ Moreover, the GDPR provides four possible ways for the further processing of personal data, namely: (1) by obtaining an informed consent,^3^ (2) if there is a European Union or Member State law that allows the secondary use,^4^ (3) a positive outcome of the compatibility assessment contained in Article 6(4) GDPR, i.e., the new processing purpose must be compatible with the original purpose for which the data were collected,^4^ and (4) the presumption of compatibility for scientific research, which means that the re-use of personal data for scientific research is considered compatible with the initial purposes, provided that appropriate safeguards are in place.^5^ The views and experience of study participants with the latter two methods were probed.

### Participants and recruitment

The study targeted the following stakeholder groups: (1) data protection officers (DPOs) or legal experts working in the pharmaceutical industry, academia, and academic biobanks involved in clinical research, (2) investigators (physicians), and (3) members of ethics committees (ECs). Stakeholders needed to be involved in clinical research, speak fluent English and be active in an EU Member State or in the United Kingdom (UK).

The survey was broadly disseminated on social media (e.g., LinkedIn, Twitter) and via the research team’s professional network, as well as through international consortia and networks, such as the Innovative Medicines Initiative (IMI) Consortium Corona Accelerated Research & Development in Europe (IMI CARE), the European Organization for Research and Treatment of Cancer (EORTC), the European Network of Research Ethics Committees (EUREC), the European Forum for Good Clinical Practice (EFGCP), and the European Association of Health Law (EAHL). Only survey participants who volunteered and shared their contact details with the research team were invited to participate in a follow-up interview. Each interview was digitally recorded and subsequently transcribed *ad verbatim* by a third party. Responses were recorded between April and December 2021. The study was reviewed and approved by the Ethics Committee Research UZ/KU Leuven (S65106).

### Analysis

Survey data were analyzed descriptively in Microsoft Excel. Percentages were calculated based on the number of respondents for each question. The sample size varied throughout the survey due to the logic applied in the survey questions and due to respondents’ drop-out.

Interview data were analyzed in accordance with the framework method (25), using the NVivo^®^ software. The Qualitative Analysis Guide of Leuven was followed (26). The coding of all transcripts was performed by one researcher, based on a working analytical framework. Although no cross-check was performed, the use of the other stages of the framework method, and the availability of existing literature to inform the coding process minimized subjective interpretation of the data.

## Results

Survey results are presented first, followed by the insights obtained through the interviews. The presentation is structured according to the main topics explored in the study.

### Demographics

#### Survey

In total, 191 participants completed the survey, of which more than half were investigators (52%, *n* = 100/191), followed by EC members (24%, *n* = 46/191) and DPOs/legal experts (24%, *n* = 45/191). The majority of respondents who characterized themselves as members of ECs were mainly physicians (33%, *n* = 13/39) and lawyers (26%, *n* = 10/39), and were involved in a local or regional EC (56%, *n* = 22/39). In addition, DPOs/legal experts were mainly working at a pharmaceutical company (19%, *n* = 6/31), a research institute (19%, *n* = 6/31), or other institutions (42%, *n* = 13/31) such as universities, consultancy companies or law firms. Out of all targeted 28 countries (EU Member States and the UK), 25 were represented in the survey. Most respondents were based in Belgium (17%, *n* = 26/152), the UK (11%, *n* = 16/152), Italy (11%, *n* = 15/152), Germany (8%, *n* = 12/152), and the Netherlands (7%, *n* = 11/152).

**FIGURE 1:**
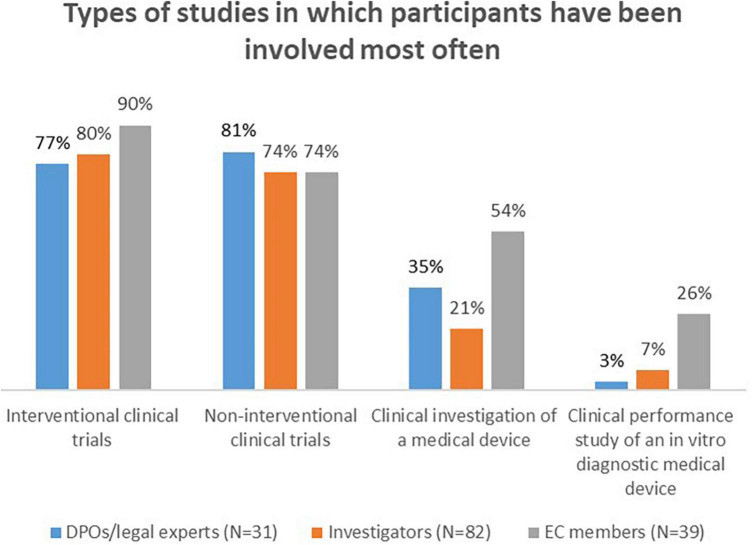
Types of studies in which survey participants have been involved most often.

Most DPOs/legal experts (71%, *n* = 22/31) were involved in international studies (on a global scale: both across the EU and outside the EU), whereas the majority of investigators (71%, *n* = 58/82) had experience with national studies, conducted in the country where they are based. EC members mostly had experience with the assessment of studies conducted in several EU countries (90%, *n* = 35/39). All stakeholder groups were mainly involved in interventional and non-interventional clinical trials (Figure 1). In addition, the majority of DPOs/legal experts (55%, *n* = 17/31) and investigators (61%, *n* = 50/82) had experience with registry-based trials. Moreover, both DPOs/legal experts (87%, *n* = 27/31) and investigators (99%, *n* = 81/82) were primarily involved in non-COVID-19 studies. More than half of the investigators (59%, *n* = 44/74) and almost half of the EC members (48%, *n* = 15/31) reported having received a formal training about the GDPR.

#### Interviews

Out of 31 volunteers invited to participate in the qualitative arm of the study, 14 agreed to be interviewed. More than half were DPOs/legal experts (*n* = 8/14), followed by EC members (*n* = 4/14) and investigators (*n* = 2/14). All EC members had a legal background. The interviewees were based in seven EU Member states and the UK and were primarily active in international clinical studies (*n* = 13/14).

### Primary (prospective) use of personal data for research purposes

#### Choice of a legal basis: Article 6(1)

All stakeholder groups were asked to indicate how often their organization relies on (in the case of DPOs/legal experts or investigators) or advises about (in the case of EC members) the use of one of the six legal bases available under Article 6(1) GDPR. DPOs/legal experts shared that their organizations use the legal bases “Consent,” “Legal obligation,” “Public interest,” and “Legitimate interests” mostly “Sometimes,” whereas “Contract” and “Vital interests” – “Never.” The majority of investigators indicated that they use all possible legal bases “Always” or “Most of the time,” whereas most EC members showed a preference for “Consent” (Supplementary Figure 1 in Supplementary material 3).

Additionally, the majority of DPOs/legal experts who were active in international studies, indicated that, most of the time, they rely on the same legal basis in each country involved in a cross-border clinical trial.

##### Interviews

The majority of interviewed DPOs/legal experts personally opposed the use of consent as legal basis in clinical trials. They remarked that although consent was the prevalent legal basis prior to the entry into force of the GDPR, the situation has since changed, especially due to the guidance provided by the EDPB. Nevertheless, most of the DPOs/legal experts reported that they still apply consent in certain situations, more specifically in cross-border trials. It was reported that some national laws make the use of consent as legal basis obligatory (i.e., Netherlands, Germany, Austria, Ireland, Poland), and that other countries have mandated the use of consent through authoritative opinions of their health authorities or ethics committees.

In addition, four DPOs/legal experts addressed the choice of a valid legal ground in cross-border studies. All of them shared that they accept what is insisted upon locally. Two experts mentioned that the divergence of legal bases in one single study complexifies it and brings more uncertainty: for the patients, for the sponsor and for the public. They opined that a uniform approach would be less costly, and it would allow better protection of the data protection rights. A varying approach, on the other hand, could potentially lead to fewer open investigational sites in the future (although none of the DPOs had stopped working with a site because of the legal basis), and it may mean a *“longer time to bring a pharmaceutical product to the market*.”

#### Choice of a special condition: Article 9(2)

Most DPOs/legal experts indicated that their organizations use “Explicit consent” about half the time, “Public interest in the area of public health” - sometimes and “Scientific research” – most of the time. In comparison, the majority of investigators shared that they always use all possible legal conditions (Supplementary Figure 2 in Supplementary material 3). The interviews did not provide additional information on this topic.

#### Ethics committees’ advice

Data protection officers/legal experts and investigators were asked whether ECs sometimes insist on the use of a specific legal basis under the GDPR for the primary use of personal data. The majority (63%, *n* = 10/16 and 76%, *n* = 22/29, respectively) responded positively, and stated that ECs would request the use of “Consent” as legal basis. EC members were also prompted to share whether they tend to suggest the use of a specific legal basis. More than half responded with “Yes” (62%, *n* = 15/24).

##### Interviews

Two of the interviewed DPOs/legal experts provided insights on this topic. In the experience of one of them, the need to base a study on consent (in the cases when this was not mandated in national law) was often due to the advice provided by ECs. According to another DPO/legal expert, they have received divergent advice from national and even regional ECs, and in *“some cases”* had to stop a study because of this. According to them, two major trends across the EU could be outlined as regards ECs approach: some ECs are very involved in data protection, whereas others do not intervene on this topic. However, no detailed mapping of EC approaches currently exists.

The members of ethics committees who partook in the interviews also discussed their views. One EC member shared that they have a strict policy in cases when a university hospital acts as sponsor/data controller, namely that they insist that the legal basis should be public interest. If another entity is the sponsor, the same interviewee acknowledged that the responsibility for choosing a valid legal ground is for the controller, and they would not insist on a legal basis. However, they would voice their concerns in writing in case they do not think the chosen legal basis is the most optimal one (for instance, when a commercial sponsor has chosen to rely on public interest).

“*We don’t think that’s a good lawful basis, but you’re the data controller and you’re responsible”* (EC member).

The same approach (voicing concerns, but not interfering) was reported by another EC member particularly opposed to the use of consent. Finally, half of the EC members who were interviewed mentioned that they prefer consent in all cases due to national sensitivities (Germany, Portugal).

#### Changes during the pandemic

The majority of survey respondents from all stakeholder groups (DPOs/legal experts: 89%, *n* = 16/18; investigators: 71%, *n* = 10/14; EC members: 80%, *n* = 12/15) reported that there was no change in the legal basis/special condition on which their organization relied during the pandemic. A few exceptions were reported, in particular related to an increased use of “Public interest” as legal basis.

The interviews provided similar results.

### Secondary (retrospective) use of personal data for research

#### Compatibility assessment

**FIGURE 2:**
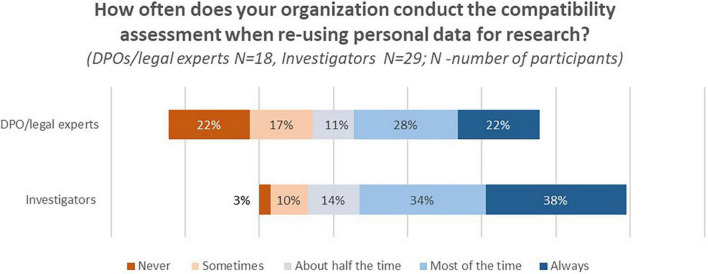
DPOs/legal experts’ and investigators’ answers to the question “How often does your organization conduct the compatibility assessment when re-using personal data for research?”

Data protection officers/legal experts and investigators were asked to indicate their experience with the compatibility assessment. The majority of respondents from both stakeholder groups indicated that they conduct the test “Most of the time” or “Always” (Figure 2).

Additionally, DPOs/legal experts and investigators had to rank **the elements of the compatibility test**.^6^ DPOs/legal experts considered the element “Any link between the purposes for which the personal data have been collected and the purposes of the intended further processing” as “Most important.” In contrast, the majority of investigators considered “The nature of the personal data” and “The existence of appropriate safeguards” as most important.

##### Interviews

The majority of interviewees shared that the most important element of the compatibility test was the link between the purpose of the primary use and the purpose of the further processing of the personal data. Additionally, one DPO/legal expert determined three so-called “levels” at which they look to determine the link, namely:

1) the research is *“necessary to understand the pharmaceutical product in a better way*,” meaning that the further processing is conducted in relation to the same pharmaceutical product which was tested in the initial clinical trial. A positive answer to this question would show the strongest connection between the primary use and the intended further processing;
2) the research is *“linked to the specific disease that was targeted in the original trial”*;
3) the research is necessary to have *“better methodology or techniques to diagnose”* the disease that was the target of the initial study.

Most interviewees discussed the importance of ethics committees in assessing the conduct of the compatibility test, in particular that ECs should further “look” at the test, as performed by the controller. Furthermore, one DPO/legal expert emphasized the need for more authoritative guidance (issued by the competent supervisory authorities) on the conduct of the different types of balancing exercises that are foreseen in the GDPR [compatibility test, legitimate interests of the controller under Art. 6(1)(e), data protection impact assessment].

#### Presumption of compatibility

**FIGURE 3:**
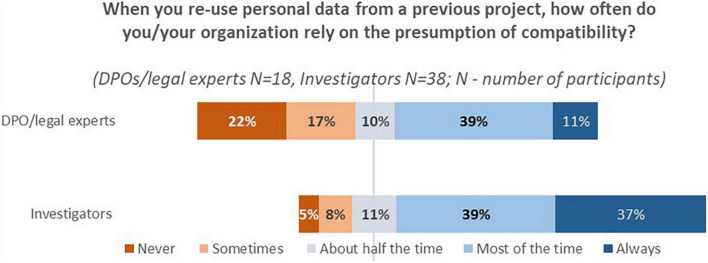
DPOs/legal experts’ and investigators’ answers to the question “When you re-use personal data from a previous project, how often do you/your organization rely on the presumption of compatibility?”

The majority of DPOs/legal experts and investigators shared that they rely on the presumption of compatibility “Most of the time” (Figure 3).

Finally, a cross-tabulation of the results showed that most respondents from both stakeholder groups do conduct the assessment even though they rely on the presumption of compatibility (DPOs/legal experts 72%, *n* = 13/18; investigators 86%, *n* = 25/29).

##### Interviews

The majority of the interviewees relied on the presumption of compatibility, as long as the research was performed in the same therapeutic area. Most of them also discussed additional conditions, such as the case when the new study is performed by *“the same researcher,”* and when it is about *the “same investigational medicinal product.”* One DPO/legal expert shared that their organization did not rely on the presumption, but instead preferred to re-consent the participants, stating that this is because they *“don’t feel comfortable with the approach”* of the regulators (i.e., lack of clarity and guidance).

Most individuals who relied on the presumption still performed the compatibility assessment, either in part or in full. As described by one DPO/legal expert, performing the compatibility test *“on top of”* the presumption of compatibility, was considered *“essential”* to ensure that there is no infringement on the rights and freedoms of individuals.

The expectations of the data subject emerged as the most important element of the compatibility test when applied together with the presumption of compatibility, particularly in view of the importance *“to be respectful to individuals”* (DPO/legal expert). One ethics committee member, who expressed a preference toward the re-consenting of data subjects *(“because it is safer for the researcher and for the patient”*), shared that in cases where obtaining consent is impossible, they would allow the reliance on the presumption of compatibility, on the condition that the researchers conduct a balancing test, included in writing in the new study protocol. Namely, that the interests of the study subject are weighed against the “*research interests.”*

Some participants shared how they practically document the fact that they rely on the presumption of compatibility. For instance, among DPOs/legal experts, some experts preferred structured approaches (such as the Nymity GDPR Compliance Toolkit^7^), whereas others relied on email exchange with the researcher, in which they confirm that the data can be reused. Ethics committee approval of the protocol of the secondary use study also emerged as an important safeguard.

#### Need for a new legal basis

**FIGURE 4:**
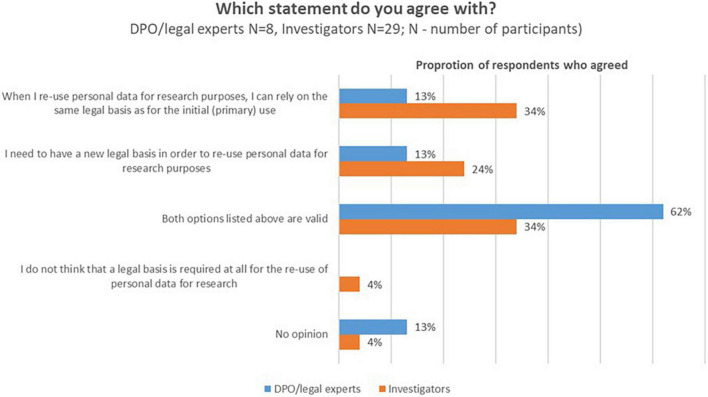
DPOs/legal experts’ and investigators opinions about the need for a new legal basis when data is processed for secondary use.

Currently, one of the most contested questions in literature and practice is whether a new legal basis should be chosen for secondary use, or whether the processing could continue to be based on the legal ground used for primary use (if specific conditions apply). DPOs/legal experts and investigators were confronted with a series of statements to gauge their view on this issue. As displayed in Figure 4, more than half of the DPOs/legal experts indicated that a legal basis is required and that they rely either on the same legal basis as for the primary use of the personal data, or on a new legal basis (62%, *n* = 5/8), while investigators expressed more diverse opinions.

##### Interviews

Few interviewees (all of whom were DPOs/legal experts) opined on this topic. According to one interviewee who relied most often on consent as the valid ground for the primary processing, the initial consent would *“most often be sufficient”* for the secondary use of the data, as well. However, another DPO/legal expert held the opposite view, namely that if one relied on consent as the basis for the primary use, it would be *“very difficult to use the [same] consent for the reuse”* of the personal data. Moreover, they reflected upon EDPB’s guidance on this topic and explained that they understand it to mean that *“you might not always be able to use the same basis”* (as for the original processing).

#### Ethics committees’ advice

In the survey, DPOs/legal experts and investigators were asked whether ECs sometimes insist on the use of a specific legal basis under GDPR for the secondary use of personal data. More than half (53%, *n* = 8/15 and 58%, *n* = 18/31) indicated “Yes.” Of those who provided examples, the majority indicated that they are advised to use “consent.” Around half of the EC members (52%, *n* = 12/23) also replied positively to the question whether they suggest what legal basis/special condition should be relied upon for secondary use of personal data in research.

#### Changes during the pandemic

The majority of DPOs/legal experts and investigators who participated in the survey indicated that during the pandemic there was no change in the way that they process personal data for secondary use (DPOs/legal experts: 84%, *n* = 16/19, investigators: 90%, *n* = 47/52).

All interviewees reported the same.

### Transparency

In the survey, all stakeholder groups were asked to share the biggest challenges they experienced when providing GDPR-related information to study participants prior to the pandemic (open-ended question). In total, *n* = 15 DPOs/legal experts, *n* = 33 investigators, and *n* = 17 EC members responded. One major common topic emerged from their answers, namely the challenge of providing clear explanation about complex topics (such as data subjects rights, the relationship between the parties involved in the processing, distinguishing between informed consent as ethical requirement and consent as legal basis under GDPR, anonymization and pseudonymization, etc.). In addition, investigators specifically addressed the limited time they have to provide information. Moreover, according to a limited number of investigators, patients are not interested in GDPR-related information, as they are already highly motivated to participate. Finally, EC members commented that, in their view, there is insufficient awareness and knowledge about data protection among researchers. According to ECs, this consequently makes providing GDPR-information from researchers to participants challenging.

Participants were also prompted to indicate whether there was any change in the challenges they experienced during the COVID-19 pandemic. For most survey respondents, there was no change (DPOs/legal experts 72%, *n* = 13/18; investigators 88%, *n* = 43/49; EC members 91%, *n* = 21/23).

#### Interviews

Interviewees were asked to share how they currently inform study participants about the processing of personal data for research for primary use. All participants reported that they comply with their transparency obligation during the process of obtaining the informed consent for participation in the study, by either including the relevant information in the patient information sheet that accompanies the ICF, in the ICF itself, or in an annex to the ICF. Some reported the use of a layered approach, namely the inclusion of a reference to an online website where more detailed information is presented.

One participant shared the approach adopted by hospitals in Belgium as regards transparency for secondary use, more specifically the use of the secure web application *mynexuzhealth*.^8^

One DPO/legal expert criticized the approach of some ethics committees with respect to the advice they provide on this topic. They reported that “*in some places, you have to state the specific GDPR article for every sentence, which for most patients is useless,”* and that other ECs are *“adamant”* that the contact details of the controller are included: *“But in a double-blinded study, if you do that, if patients contact the sponsor, they unblind themselves.”*

Additionally, several interviewees discussed that, when pseudonymized health data is shared by their organization to another controller, they impose contractual obligations to the new controller, pursuant to which the new controller would have to inform them about any future uses of the data. Their organization, as original controller and as having an established connection with the data subjects, would then inform the data subjects about the new processing of their personal data.

Finally, interviewees were asked to provide their opinion on the ideal way to inform study participants about the processing of their personal data in clinical research. All experts emphasized the need for shorter data protection and privacy notices than the current standard practice. In addition, they all discussed the necessity of phrasing the written information into a “clearer,” more “use-friendly” and “lay” language, as well as the importance of using visual elements (such as cartoons, infographics, and flowcharts). Several interviewees opined that electronic means would be useful as well. One DPO/legal expert additionally discussed the need to add an oral discussion with a legal expert, in addition to the oral explanation that is typically provided by the investigator.

### Communication with ethics committees

#### Role for ethics committees under the General Data Protection Regulation

DPOs/legal experts and investigators were asked whether, according to them, the GDPR establishes a special role for ethics committees when it comes to the way personal data is processed for scientific research. More than half (DPOs/legal experts: 63%, *n* = 10/16; investigators: 53%, *n* = 25/47) responded negatively. Participants who opined that ECs have a special role under GDPR were prompted to share their reasons in an open box. The main topics mentioned by DPOs/legal experts were: (1) Recital 33 GDPR^9^, and (2) that ECs evaluate the “*ethical merit of the research initiative*,” and they should protect the rights and the freedoms of the study participants, which include data protection and privacy.

DPOs/legal experts and investigators who responded negatively to the previous question, were asked to share whether ECs should have a special role under GDPR. The majority (DPOs/legal experts: 70%, *n* = 7/10; investigators: 80%, *n* = 20/25) responded “No,” citing the following reasons: (1) the responsibility of data protection authorities, (2) the responsibility and accountability of data controllers, (3) the view that ECs are “*prone to inconsistency and limited understanding of the practical application of legal requirements in the context of the highly regulated environment within which the pharmaceutical industry operates*.”

##### Interviews

Most interviewees were opposed to ECs having a role in the determination of core choices that are under the responsibility of the controller (e.g., the choice of a legal basis). However, the experts discussed that as, in reality, ECs do intervene, it would be useful to clarify their role in data protection in an official manner. Moreover, study participants emphasized the importance of making the inclusion of a trained data protection expert in ECs mandatory. As reported by several interviewees, the level of data protection knowledge among ECs in the EU “may be quite different,” creating additional difficulties in interactions and discussions.

*“In many cases, they [ECs] may not actually be fully up to speed with the positions taken by the EDPB, and so on”* (DPO/legal expert).

Finally, EC members who took part in the interviews emphasized that it is important for them to check that the personal data of study participants is well-protected, as part of their responsibility with respect to the general protection of research subjects. However, one EC member opined that the assessment of data protection questions *“takes a lot of time for the ethics committees*,” and *“that’s maybe not what the ethics committees should mainly be doing*,” as they must focus on the *“actual physical risk to the patients”* (in interventional studies).

#### Ethics committees’ comments in relation to the General Data Protection Regulation

In the survey, the majority of DPOs/legal experts and investigators (77%, *n* = 10/13 and 66%, *n* = 27/41, respectively) indicated that they receive comments regarding GDPR-related text in study protocols. In total, 9 DPOs/legal experts and 20 investigators provided examples in an open box. Three main themes were identified. First, ECs mandate the inclusion of text in the study protocol that could be challenging for the sponsor (e.g., a detailed summary of further processing which can be unknown at the time of the primary collection), or that could go against the applicable legal rules (such as mandating that the contact details of the sponsor are shared with the study participants; imposing the legal basis on which the processing of personal data should be based). Second, comments related to terminology and readability of the GDPR-related text, for example criticism as regards the complex phrasing of the relevant text. Third, ECs impose restrictions related to data storage periods or data transfers.

All stakeholder groups were asked whether ECs advise on the use of specific techniques for anonymization or pseudonymization of personal data. The majority of DPOs/legal experts shared that this does not occur (84%, *n* = 11/13), whereas around half of the investigators (52%, *n* = 21/40) had experienced such advice. EC members’ self-assessment registered 74% (*n* = 17/23) negative responses.

Finally, DPOs/legal experts and investigators had to share whether, in their view, their studies may become non-compliant with the GDPR if they follow some of the comments they receive from ECs. The majority (60%, *n* = 6/10 and 83%, *n* = 20/24, respectively) did not think so. Of those who responded “yes” (40%, *n* = 4/10 and 17%, *n* = 4/24, respectively), DPOs/legal experts reported that they try to challenge the comments.

The interviews did not present additional information on this topic.

### Main challenges prior to and during the pandemic

Prior to the COVID-19 pandemic, the majority of DPOs/legal experts and investigators considered that they experience challenges when it comes to “Lack of legal harmonization” “most of the time” and “about half the time,” respectively. In comparison, according to EC members’ observations through working with researchers, the most challenging topics were “Choice of legal basis for primary use,” “Secondary use of personal data,” and “Providing information to study participants.”

When asked to rate the main challenges again, this time during the pandemic, no significant change was registered in the views of DPOs/legal experts and EC members.

Finally, EC members were asked to share their view on whether commercial and academic sponsors experience different challenges in relation to GDPR compliance. More than half (68%, *n* = 13/19) reported to have observed differences, and one main topic emerged from the examples they provided, namely that commercial sponsors are better prepared due to better qualified personnel and more financial resources to invest in data protection compliance and training.

#### Interviews

Interviewees were asked to elaborate their views on this topic. The majority (*n* = 11/14) discussed the challenge presented by the lack of legal harmonization. According to most, the main problem lies at the level of interpretation of the GDPR, as well as with respect to the divergencies in the national implementation of the regulation. Furthermore, investigators in particular reported that the lack of a harmonized approach fostered the proliferation of contradictory advice given by ethics committees. An example was given by one investigator, namely that the same study was not approved by the ethics committee of the research hospital where he is employed, but it was approved in a different hospital, in the same city.

None of the interviewees observed changes in the main legal challenges during the pandemic. As put by a DPO/legal expert: *“The GDPR does not change because of the pandemic, it is people’s perceptions.”* The pandemic had implications with respect to the practical conduct of clinical studies. In particular, interviewees noted the following difficulties: (1) recruitment and in-person visits, (2) managing studies with staff working remotely, and (3) remote monitoring.

Finally, interviewees were asked about their views on the ideal way to address the main challenges related to compliance with the GDPR. Half of the study participants emphasized the need for a harmonized interpretation of the regulation. More specifically, it was voiced that more collaboration between data protection authorities is necessary, as well as specialized EDPB guidelines on health research. Most participants did not see value in “re-opening” the GDPR and/or the adoption of new regulations:

*“We just need clarification and standardization mechanics to ensure that GDPR applies to clinical research”* (DPO/legal expert).

## Discussion

### Study results

This mixed-methods empirical study is among the first to investigate the experience of key EU and UK clinical research stakeholders as regards compliance with data protection rules before and during the COVID-19 pandemic. The results of this exploratory study provide a snapshot into the real-life practices and beliefs in relation to the application of the GDPR in clinical research. In addition, to the best of our knowledge, this study is the first attempt to explore how ECs understand their role in data protection and how other stakeholders address ECs’ comments related to GDPR compliance. The study showed that the lack of legal harmonization remains the biggest challenge in the field. Moreover, although the majority of respondents were opposed to ECs having a role in the determination of core choices that are under the responsibility of the controller, experts seemed to agree that it would be useful to clarify their role in data protection in an official manner. Last, but not the least, the findings of this study are relevant to non-pandemic situations, as most of them are not specific to COVID-19, but were only made more apparent and exacerbated in the context of the pandemic.

#### Primary (prospective) use of personal data

##### Choice of a legal basis under Article 6(1) General Data Protection Regulation

The preference shown by DPOs/legal experts was mostly in line with the advice previously given by the EDPB and the European Commission (24, 27). However, although both the EDPB and the European Commission have discouraged the use of consent as a legal basis (due to the challenge for it to be considered “freely given” in the meaning of the GDPR, because of the imbalance of power between the participants and the sponsor/investigator), surveyed DPOs/legal experts reported that they use consent seemingly as often as other suitable legal grounds. Interviews shed more light on the reasons why. In particular, this is linked to national laws that mandate the use of consent as legal basis, as well as health authorities and ethics committees that insist on it (4, 12).

It is not the aim of this publication to weigh and discuss the application of all suitable legal grounds for primary use of personal data in research, as this is already the topic of scholarly debate (4, 5, 12, 28, 29). Nevertheless, three general observations are merited.

The **first issue** pertains to the **collected evidence that ethics committees tend to advise on the appropriate legal basis** (62% of surveyed EC members, 73% of DPOs/legal experts, 76% of investigators). In case the EC would impose its view [which could be perceived to occur at least to some extent, based on the results of this exploratory study as well as past anecdotal evidence (11, 12)], this would be in direct contradiction with the GDPR accountability principle.^10^ Pursuant to the accountability principle, data controllers are responsible for compliance with the data protection law and should be able to demonstrate this. Thus, identifying an appropriate legal basis should be the prerogative of the controller. Even if the EC advice is not imposed, a clear delineation of the role and responsibilities of ethics committees when it comes to providing data protection compliance suggestions is still necessary (see also the subsection “Role for ethics committees” below).

The **second issue** pertains to **the choice of a legal basis in cross-border studies**. Although many surveyed DPOs/legal experts opined that most of the time they rely on one legal ground for the processing of data in the scope of the same study across Europe, the results must be discussed in view of the evidence that identifying an appropriate legal basis is influenced by divergent national laws on the one hand (4) and by differing advice (from DPAs and ECs), on the other. It could be expected that the legal basis seen as most suitable would vary across EU Member states, and harmonization in the scope of a cross-border study (i.e., the use of one single basis) would be hard to achieve in practice. Using different legal bases in the scope of one cross-border clinical trial potentially creates inequalities between patients from different countries, as different legal bases come with diverse application of the rights of the data subjects (12, 30). Based on the responses of interviewees, as well as the reflections shared in relevant scholarship, it seems that at present the main way to rely on one legal basis in a cross-border study would be through consent. This goes against the advice provided by the EDPB and the Commission. Moreover, literature has identified many circumstances in which consent would not be the most appropriate, or even valid, basis (4, 12, 29, 31–33). Additionally, the Commission’s assessment study showed that “*the understanding and application of consent can vary significantly across Member States*,” which underlines the need to further discuss and clarify the notion of consent under the GDPR (4).

Finally, it must be underscored that **the role of the cross-border clinical research participants as data subjects** appears to be insufficiently investigated so far, even though the clinical research decision-making process is progressively influenced by a Europe-wide trend toward a patient-focused approach in the design of clinical studies. In this trend, the concepts of patient empowerment – which aims to ensure that patients’ needs and priorities in healthcare and research are identified and met – and the intertwined notion of data control, hold center stage (34–39). While the concept of “data control” appears to be central to the GDPR,^11^ and is frequently invoked in EU policy initiatives and legal scholarship (18, 40), a clear definition of this notion does not exist at present. Concerns have been voiced regarding the question of how to reconcile the GDPR objectives to (1) protect individuals for the processing of data related to them and (2) to increase (personal) data flow/(re)use of personal data (40). Two main stages for the exercise of data control in the health research cycle could be identified: (1) at the stage of data collection, and (2) after personal data has already been collected (40–43). Related to the first stage (data collection), it is well known that data control historically places much emphasis on consent. However, the challenges related to consent were already alluded to above. Related to the second stage (after data collection), the emphasis is put on data subjects’ rights afforded under the GDPR, especially on data portability, right of access, right to withdrawal of consent and the right to object. However, previous studies (conducted among citizens) have shown that individuals are aware of and exercise their rights only to a limited extent (44). As data control and data subject/patient empowerment are currently increasingly in the focus of important new policy initiatives (15–18), an in-depth investigation and comparison between the application of the different legal bases with regard to the level of data subjects’ control and the exercise of data subjects’ rights in (cross-border) clinical research appears to be highly necessary.

##### Choice of a special condition under Article 9(2) General Data Protection Regulation

Survey results showed no major difference between the EDPB and Commission advice (24, 27) on the one hand and choices by DPOs/legal experts on the other hand regarding the frequency of use of the special conditions under Article 9(2) GDPR. However, a more detailed discussion is out of the scope of this paper, as the format of the survey did not allow participants to show the various ways they could “couple” Article 6 and Article 9, and the interviewees did not discuss their answers with respect to Article 9 in more detail.

#### Secondary (retrospective) use of personal data

The Commission’s “Assessment of the EU Member States’ rules on health data in the light of the GDPR” investigated the EU legal framework for secondary use of health data for scientific research (4). The report focused on the legal bases for secondary use, which it defined as “the re-use of health data that were collected initially in the context of *providing care*.” Unlike the Commission study, our survey aimed to investigate the experience of key research stakeholders as regards the conduct of the compatibility assessment [Article 6(4) GDPR] and the reliance on the presumption of compatibility [Article 5(1)(b) GDPR]. Moreover, participants in our study mostly discussed the reuse of personal data that was initially collected in the context of *clinical research*. Therefore, this publication could be considered as complementary to the Commission assessment report. Additionally, it is merited to compare our results with the reflections presented in the Commission report. Most crucially, the Commission’s assessment concluded that “*there are different rules and regulations governing access to health data both within and between Member States, which impact researchers both in the context of in-country and cross-border research*,” thus affecting the availability and accessibility of health data. Our study re-confirmed this finding, with the addition of drawing further attention to the complicated role of ethics committees.

##### Compatibility assessment

Most DPOs/legal experts and investigators who participated in this study reported that they perform the assessment, which seems to suggest that the understanding of this tool, as well as the preference of using the assessment (over other tools, such as re-consenting the data subjects) might be relatively high in our sample across the EU and UK.

The GDPR does not ascribe different importance to the various criteria of the compatibility test. However, the Article 29 Working Party^12^ (WP 29) has previously noted that an inherent characteristic of such a multi-factor assessment is that *“deficiencies may in some cases be compensated by better performance on other aspects*” (45). The WP 29 discussed this possibility for compensation with respect to the element appropriate safeguards. In the scope of the survey, however, stakeholders ranked other elements as most important – namely “*the link between the purposes”* for the primary and secondary use of the personal data (DPOs/legal experts) and the “*nature of the personal data”* (investigators).

Based on the interview discussions, the “link between the purposes” appears to be preferred specifically in the context of using the compatibility test on a stand-alone basis, i.e., when the researchers do not rely on the presumption of compatibility. The WP 29 summarized that: “*the greater the distance between the purposes of collection and the purposes of further processing, the more problematic this would be*” (45). However, the question about whose perspective should be definitive in the assessment of said distance has not been substantially considered in the GDPR and jurisprudence (46). According to Zuiderveen Borgesius and Hallinan, the difference should be looked at “*from the perspective of all relevant stakeholders in processing – controller, processor, data subject, and Data Protection Authority*,” with more weight given to the perspective of the data subject (46).

Furthermore, the interviews highlighted that some research stakeholders find it important that ethics committees assess the compatibility test after it has been performed by the controller. In fact, ethics committee reviews of the test appear to already occur across Europe, to the extent that some interviewees have voiced the wish for authoritative guidance (issued by data protection authorities) on the conduct of the compatibility test and the role of ethics committees in its assessment. The Commission report discussed “*the variable judgments of ethics committees in considering the compatibility of research applications to reprocess data with the original trial protocols”* as a barrier to the reuse of health data (4).

##### Presumption of compatibility

Pursuant to Article 5(1)(b) of the GDPR, further processing of personal data for scientific research purposes shall not be considered incompatible with the initial purposes, as long as the processing is subject to appropriate safeguards [Article 89(1) GDPR]. These safeguards shall ensure that technical and organizational measures are in place to guarantee respect for the principle of data minimization (e.g., pseudonymization). Most DPOs/legal experts and investigators who participated in the study relied on the presumption of compatibility, and most of them still conducted the compatibility assessment (in full, or partially).

At first glance, it might appear that Article 5(1)(b) creates a legal fiction, i.e., that all processing for scientific research purposes would automatically satisfy the compatibility test in Article 6(4) GDPR. However, according to scholarship, the consequence of the presumption is to make processing for scientific research purposes “*privileged”* and not subject to the barriers that the purpose limitation principle creates for secondary use of personal data (46). According to Verhenneman et al., this favorable position for scientific research is created by “*limiting the compatibility assessment to an assessment of the appropriate safeguards*” (33). The emphasis on conducting an assessment of the appropriate safeguards is echoed by other scholars as well (46–48), and it is supported by the European Data Protection Supervisor (EDPS), which stated that: “*The presumption is not a general authorization to further process data in all cases for historical, statistical or scientific purposes. Each case must be considered on its own merits and circumstances. But in principle personal data collected in the commercial or healthcare context, for example, may be further used for scientific research purposes, by the original or a new controller, if appropriate safeguards are in place”* (49).

It is of interest to observe, then, that the interviewees who reported that they conduct a partial compatibility test focused on the expectations of the data subjects, and not on the appropriate safeguards, in contrast to scholarly and institutional advice. The expectations of the data subjects are not part of the five elements of the compatibility test, specified in Article 6(4), but are mentioned in the key Recital 50 GDPR: “In order to ascertain whether a purpose of further processing is compatible with the purpose for which the personal data are initially collected, the controller, after having met all the requirements for the lawfulness of the original processing, should take into account, *inter alia*: (…) the context in which the personal data have been collected, *in particular the reasonable expectations of data subjects based on their relationship with the controller as to their further use”* [authors’ emphasis]. Such reasonable expectations of the patient and society at large should also be seen in the context of regulatory instruments, in particular the Council of Europe Recommendation No. R(97)5 on the protection of medical data (50), later replaced by Recommendation CM/Rec(2019) (51). R(97)5 allowed secondary use of health-related data by the health-care professional – treating physician for ‘own medical research’ provided this was transparent and patients could opt-out and insofar domestic law would provide for the legal ground and/or safeguards.^13^ CM/Rec(2019) eventually even broadend “own medical research” by the health care professional to also by “other scientists in other disciplines” insofar appropriate safeguards are in place. The context in which the above mentioned instruments allowed for such secondary use for research would merit further analysis, for example also with regard to the responsibility for this secondary use, to better understand how the notion of “reasonable expectations” may have evolved over the last decades.

Furthermore, participants who performed the full compatibility test appear to see it as an additional safeguard. Such a view is in line with the advice previously provided by the EDPS (49).

The European Data Protection Board previously stated that “*for the time being, the presumption of compatibility, subject to the conditions set forth in Article 89, should not be excluded, in all circumstances, for the secondary use of clinical trial data outside the clinical trial protocol for other scientific purposes”* (24) and acknowledged the processing of personal data for secondary use will require further guidance from the EDPB in the future. Such guidance is indeed urgently needed, as the study results reflect.

#### Transparency

Data subjects have the right to be informed about why their data is processed, how it will be used and by whom.^14^ Information shall be provided “*in a concise, transparent, intelligible and easily accessible form, using clear and plain language*.^15^“ For the purpose of our study, we aimed to investigate how key research stakeholders comply with their obligation of transparency toward data subjects, primarily in the context of primary use of personal data. The study suggests that information is most often provided in writing, as part of the documentation that accompanies the informed consent form for participation in the study, and that it is orally explained by the investigator. Including the GDPR notice as part of the ICF could provide a ground for confusion in cases where the legal basis “consent” is relied upon for the processing of personal data.

The biggest transparency challenge that emerged from the study, was that researchers currently find it difficult to be clear and concise when providing GDPR-related information to participants. Indeed, there has been much debate about the problem of communication of data protection and privacy policies, usually written “*by lawyers for lawyers”* in a complex language riddled with legal technical terms (52). Although non-user-friendly information has been usually discussed with respect to online platforms (53, 54) our study results seem to suggest that the issue is prevalent in clinical trials as well.

Participants focused on several suggestions for ways to provide information, in particular visuals/flowcharts, layered approaches, and electronic means. All of these suggestions are allowed under the GDPR.^15^ An appropriate way forward, in the view of the authors of this paper, would be to explore the use of legal design methods and approaches in the drafting of data protection and privacy notices for clinical trials, as well as for the preparation of training materials for investigators and ethics committees (55, 56). Legal design is a “*discipline that combines law, technology, and design to create user-friendly legal documents and, more in general, make the legal system closer and more accessible to people. (*…*) The concept of legal design draws on design thinking, a methodology to solve problems in a creative and human-centric way”* (52).

Finally, it is of interest to discuss the finding that ECs sometimes insist that the contact details of the controller (in most cases, the sponsor of the trial, be it academic or commercial) are included in the notice. Such recommendation is in line with the provisions of the GDPR [Articles 13(1)(a) and 14(1)(a)]. However, the possibility for data subjects (patients) to contact the sponsor directly is not ideal, as in this case their identity would become known to the sponsor. In the clinical trials’ field, the sponsor (i.e., the individual, company, institution or organization which takes responsibility for the initiation, for the management and for setting up the financing of the clinical trial^16^) is typically considered the controller as regards the processing of personal data. The investigator (i.e., the ‘individual responsible for the conduct of a clinical trial at a clinical trial site^17^) is the processor (the existing divergencies in national interpretations about the roles of controller and processor in clinical research notwithstanding). The Guideline for Good Clinical Practice of the International Council of Harmonization (ICH GCP) establishes that the sponsor receives only pseudonymized (key coded) data concerning study subjects (Principles 1.58 and 5.5.5.), and that the identification key rests with the investigator. The sponsor does not have the right to obtain the key code from the investigator and cannot know the identity of the study subjects. All relevant information about the clinical trial (including data protection and privacy notices) is provided to the patient via the investigator (treating physician). More guidance is needed as to how to protect the patients’ identity from the sponsor, while at the same time respecting their rights as data subjects. Interestingly, the most recent EDPB Guidelines 01/2022 on data subject rights – Right of access, did not include any examples from the field of scientific research in general, and clinical research in particular. The Guidelines aim to clarify the responsibility of the controller when the right of access is exercised by data subjects (57), and without the inclusion of relevant clinical trials examples, they could foster the interpretation that sponsors are in all cases obliged to be in direct contact with study participants, and thus to know their identity (58). A welcome way forward would be the inclusion of an example in the Guidelines, suggesting that whereby patients wish to contact the sponsor, they should do so through the hospital site.

#### Role for ethics committees in data protection advice

The majority of surveyed DPOs/legal experts did not think that ECs should have a special role dedicated to data protection. This was, however, nuanced in the interviews. In particular, although the majority of interviewees were against ECs imposing their views on key questions that are the responsibility of the data controller (such as the choice of an appropriate legal basis), they did agree that it would be useful to have an official clarification of the ECs role in data protection. The survey results suggest that ECs tend to advise on a broad range of GDPR-related topics, although sometimes they do not have the required training.

In our view, ethics committees could fulfill a role when it comes to risk assessments foreseen under the GDPR, as a risk-based approach is firmly embedded both in clinical research^18^ and GDPR.^19^ The clinical trial sponsor must identify, evaluate and control (i.e., reduce and mitigate) the risks posed by the research, and the rights, safety and well-being of trial participants must always prevail over the interests of science and society.^20^ The main responsibility of ethics committees is to protect potential participants in research, thus part of their independent review is the identification and weighing of the risk/benefit ratio to protect the participants. Research risks are not limited to possible physical harm, but can also include psychological, social, legal and economic ramifications. If the risk/benefit ratio is not optimal, an ethics committee may provide a conditional decision, including suggestions for revision (59). The risk assessment conducted by the sponsor and by the ethics committee is not a simple exercise, but includes both quantitative and qualitative evaluation and requires proper training.

Data protection – and the GDPR in particular – has a close relationship with ethics. The application of the legislation is not merely a technical exercise but always requires judgement. For instance, Hijmans and Raab highlight that this is at the core of processing based on the legitimate interests of the controller [Article 6(1)(f) GDPR] (60). To rely on Article 6(1)(f) GDPR, the controller must perform a balancing test, and WP29 has provided a set of criteria. The EDPB’s view (albeit in a different context) is that the most decisive criterion should be the intensity of interference that the processing of data poses for the rights and freedoms of the individual (61). Similarly, in the context of the compatibility test, scholarship agrees that the concept of “compatibility” includes an element of risk containment: “*further processing must not result in a substantively higher risk than the initial lawful processing if it is to qualify as ‘compatible”* (62). Furthermore, Meszaros and Ho argued that the “*data controller should conduct [the compatibility] test based on EU level guidelines, and the results of it should be reviewed by relevant authorities*,” in particular the data protection authorities as they are responsible for enforcing the requirements of the GDPR, but “*with the help of authorities or related* ***ethics committees responsible for scientific research**”* [authors’ emphasis].

With their role and experience in performing risk/benefit assessments for research, ethics committees may have the expertise to advise on achieving adequate balancing in the various risk/benefit assessments foreseen in the GDPR, assuming that the composition of these ethics committees is sufficiently balanced, and that they are appropriately trained in data protection.^21^ Even more importantly, the new CTR states that laypersons, in particular patients and patient organizations, should be involved in the composition of ethics committees.^22^ This further guarantees the respect of data subjects’ fundamental rights and interests. However, ethical standards are not harmonized at EU level, and national and local ethics committees may show substantial differences when reviewing the compatibility assessment, or the test under Article 6(1)(f) GDPR, which would create difficulties for cross-border clinical trials. Harmonization initiatives in the field of ethical standards, although nascent, are starting to appear. Most notably, this is the aim of the European Network of Research Ethics Committees, funded and supported by the Commission (63). At national level, there is the Nordic initiative addressing the development of a joint Nordic electronic information portal on ethics committees’ approval.^23^

It is subject to further academic investigation and policy discussion to determine whether the delineation of the ethics committees’ role and responsibility for data protection review must be provided via a change of the hard law (e.g., the GDPR, or the applicable clinical trials legislation), through guidance issued by the European Data Protection Board or any other international authority, or through soft law (e.g., a code of conduct under Article 40 GDPR).

#### Main challenges with respect to General Data Protection Regulation compliance

Surveyed DPOs/legal experts reported that the main challenge was the lack of legal harmonization, particularly in the case of cross-border studies. The lack of harmonization was discussed in three main respects: (1) the divergent national implementation of the GDPR, (2) the unharmonized interpretation of the GDPR, and (3) the contradictory advice provided by ethics committees (national and local ones). This finding is fully supported by key literature on the topic (1–12, 21, 34, 41, 48). As regards the way forward, interviewees emphasized the need for a harmonized interpretation of the regulation, instead of the introduction of changes into hard law. The European Commission Assessment report also previously concluded that there is a high interest in “*further EU level action to create a more level, and above all more understandable, playing field for research using health-related data”* (4). However, in contrast to the responses collected in our study, the majority of stakeholders who participated in the Commission report saw a need for “EU level legislation.” The Commission is putting a lot of promise on several upcoming laws – such as the Data Governance Act, the Data Act, and the regulation of the European Health Data Space. However, there is a risk that the new legislative initiatives could further exacerbate the lack of harmonization in the field (19).

#### The impact of the COVID-19 pandemic

The majority of survey participants reported that the pandemic did not change their strategy as regards GDPR compliance on any of the discussed key topics, but the crisis further highlighted the deficiencies of the existing framework and the need to foster harmonization. This finding is echoed in literature and there is a consensus that the crisis provided valuable lessons for the EU and national legislators (1, 64). For instance, the authors of a literature review on the GDPR, COVID-19 and the ethical considerations of data protection concluded that “*given the lessons learned* [from the pandemic], *there is a clear and distinct need for a harmonized and collective effort and approach to global research. The authors therefore recommend further review and research to firstly ensure that an understanding of the state of the art in data protection during the pandemic is maintained and secondly support the call that has been expressed for a common multinational database that would support a GDPR and data protection compliant effort into global research”* (64).

### Future research

Several key avenues for future research were identified in this study. First, a detailed mapping of the GDPR-related advice provided by ethics committees in the EU, as well as an exploration into the possible role of ethics committees in data protection is highly necessary. This would also be a meaningful follow-up of similar efforts conducted during the time of application of the Data Protection Directive (95/46/EC), such as the PRIVIREAL project (65). This exploratory study also opens the ground for further, broader interdisciplinary research on the topic of GDPR compliance in clinical research. As an example, it is important to unravel and compare the practices for secondary use of data that were initially collected in different contexts. So far, the emphasis has mostly been put on the reuse of data collected in the context of providing care. However, more elucidation is needed about the reuse of data initially collected for research, as well as reuse conducted by the initial controller compared to reuse conducted by a new controller. Finally, the perspectives of the data subjects (study participants), are still mostly missing from the scholarly debate. It is important to elucidate empirically the patients’ awareness and knowledge about the GDPR, their experience with exercising data subject’s rights, as well as their understanding of the notion “data control” and preferences toward various data control tools (such as consent or data subjects’ rights). A valuable initiative in this regard is the ongoing project “Healthy Data,” funded by the Commission (66). The project aims to collect citizens and patients’ views on health data secondary use and sharing, and on the role that they would like to play in the management and use of their related health data, as well as to increase citizen awareness, engagement and empowerment on the topic.

### Strengths and limitations

The mixed methods design allowed us to acquire a broad understanding of the issues at hand within the project’s limited time and budget. Evidence collected through interviews cannot be generalized (by nature) and the limited survey sample is not representative, but the answers of participants in this exploratory study should be perceived as a valuable indication of the current understanding of the GDPR by key clinical research stakeholders, as well as the main challenges they experience. Additionally, this is the first exploratory empirical study that collected insights about the role of ethics committees in data protection.

The study strived for equal representation of the EU countries and the UK, and the stakeholder groups involved. Despite the high overall response rate, balanced representation was not fully achieved. The lower response rates for specific stakeholder groups could be explained by a lack of interest to participate and/or lack of interest in the topic. The stakeholder representation rate may also reflect the survey dissemination strategy, which involved the professional networks of the researchers involved in the study.

## Conclusion

This study empirically elucidated numerous key issues related to GDPR compliance in the context of (cross-border) clinical research. Survey and interview data showed that the lack of legal harmonization remains the biggest challenge in the field. The insufficient harmonization is evident not only at the level of the interplay of key EU legislative acts and as regards the national implementation of the GDPR, but also when it comes to interpretation at local, regional and company level. Moreover, the contested role of ethics committees in data protection was further explored and possible ways forward for its further normative delineation were discussed, such as an explicit clarification of the domain in which they shall operate. The empirical results also showed that the pandemic as such did not bring additional legal challenges with respect to data protection for the respondents. Although practical challenges (mainly related to the provision of information to patients) were present due to the globally enacted crisis measures, the key problematic issues as regards (cross-border) health research, interpretations of the legal texts and compliance strategies remained largely the same.

## Data availability statement

The datasets presented in this article are not readily available because of legal and ethical constraints. Study participants did not provide consent for the sharing of interview transcripts and survey questionnaires with parties other than the researchers. The anonymized survey dataset can be made available to other researchers only upon request, ensuring that the information is not used for secondary data analysis without the prior consent of the research team who conducted this original study. The interview transcripts cannot be made available, to make sure that the confidentiality and privacy of the interviewees is preserved. Requests to access the datasets should be directed to TL-S, teodora.lalova@kuleuven.be.

## Ethics statement

The studies involving human participants were reviewed and approved by the Ethics Committee Research UZ/KU Leuven (S65106). The patients/participants provided their written informed consent to participate in this study.

## Author contributions

TL-S, EDS, and IH designed the study. PV, EK, SL, AN, GV, J-JD, and RS further discussed the design of the study. TL-S analyzed the data and drafted the manuscript. PV, EK, SL, AN, GV, J-JD, RS, PB, JM, and IH thoroughly reviewed the manuscript. All authors approved the final version of this manuscript.

## Footnotes

1 De Sutter E, Lalova-Spinks T, Borry P, Valcke P, Kindt E, Negrouk A. et al. Rethinking informed consent in the time of COVID-19: an exploratory survey. Accepted to Frontiers in Medicine 2022.

2 Pursuant to the purpose limitation principle, see Article 5(1)(b) GDPR: “*Personal data shall be collected for specified, explicit and legitimate purposes and not further processed in a manner that is incompatible with those purposes; further processing for archiving purposes in the public interest, scientific or historical research purposes or statistical purposes shall, in accordance with Article 89(1), not be considered to be incompatible with the initial purposes (‘purpose limitation’).”*

3 Recital 50, Article 6(4) GDPR.

4 Article 6(4) GDPR.

5 Article 5(1)(b) GDPR.

6 Article 6(4) GDPR “(*…*) (a) ***any link*** *between the purposes for which the personal data have been collected and the purposes of the intended further processing;* (b) ***the context*** *in which the personal data have been collected, in particular regarding the relationship between data subjects and the controller;* (c) ***the nature of the personal data**, in particular whether special categories of personal data are processed, pursuant to Article 9, or whether personal data related to criminal convictions and offences are processed, pursuant to Article 10;* (d) ***the possible consequences*** *of the intended further processing for data subjects;* (e) *the existence of* ***appropriate safeguards**, which may include encryption or pseudonymisation.”*

7
https://info.trustarc.com/Web-Resource-2019-01-19-Nymity-GDPR-Compliance-Toolkit_LP.html

8 via the application, patients receive access to their medical records, including all active and completed clinical studies. At the moment, 37 research institutions are part of the nexuzhealth system. More information available at: https://assets.uzleuven.be/files/2021-11/701200_v112021_mynexuzhealth_access_to_your_medical_record.pdf; https://nexuzhealth.com/nl/zorginstellingen.

9 Recital 33 GDPR: “*It is often not possible to fully identify the purpose of personal data processing for scientific research purposes at the time of data collection. Therefore, data subjects should be allowed to give their consent to certain areas of scientific research when* *in keeping with recognized ethical standards for scientific research*. *Data subjects should have the opportunity to give their consent only to certain areas of research or parts of research projects to the extent allowed by the intended purpose”* [authors’ emphasis].

10 Article 5(2) GDPR.

11 Recital 7 GDPR: “*(*…*) Natural persons should have control of their own personal data. (*…*)”*

12 Predecessor of the European Data Protection Board.

13 Article 12(3) R(97)5.

14 Article 12, 13, 14 GDPR.

15 Article 12 GDPR.

16 Article 2(14) Clinical Trials Regulation (EU) No 536/2014 (CTR).

17 Article 2(15) CTR.

18 Principle 5 of the International Council on Harmonization of Technical Requirements for Registration of Pharmaceuticals for Human Use (ICH) Guideline for Good Clinical Practice E6 (R2), available at: https://www.ema.europa.eu/en/ich-e6-r2-good-clinical-practice.

19 Article 24 GDPR.

20 Principle 2.3 of the International Council on Harmonization of Technical Requirements for Registration of Pharmaceuticals for Human Use (ICH) Guideline for Good Clinical Practice E6 (R2), also e.g., Article 3 of the Additional Protocol to the Convention on Human Rights and Biomedicine (Oviedo Convention), concerning Biomedical research; Principle 8 of the World Medical Association – Declaration of Helsinki.

21 Since 2021, Ireland provides an interesting example in this regard. More specifically, the Data Protection Risk Assessment (DPIA), which is foreseen in the GDPR as mandatory for studies that are deemed “high risk,” must be submitted to the EC together with the application for research ethics review. See National Office for Research Ethics Committees. Guidance on data protection for research purposes for applicants, 6 September 2021, available at: https://www.nrecoffice.ie/wp-content/uploads/NREC-Guidance-on-data-protection-and-data-sharing-Final.pdf.

22 Recital 18 CTR: *“(*…*) When determining the appropriate body or bodies, Member States should ensure the involvement of laypersons, in particular patients or patients’ organizations*.”

23 The initiative is part of the 3-year priority project Nordic Council, “*Nordic research collaboration for better health.*”
