# Supplementary material for: Challenges related to data protection in clinical research before and during the COVID-19 pandemic: An exploratory study
Source: Front Med (Lausanne). 2022 Oct 10;9:995689. doi: 10.3389/fmed.2022.995689 (PMC9589288; doi:10.3389/fmed.2022.995689)
Supplement: Supplementary file 1 [file Data_Sheet_1.DOCX]

Supplementary Material 1

# Survey questionnaire

*Table of Contents*

Introduction to the survey ……………………………………………………………………........... 2

Introductory questions for all stakeholder groups …………………………………………............... 4

Questions for data protection officers and legal experts ………………………………………........ 7

- Introductory questions ………………………………………………………………………. 7
- Part I. Compliance with the General Data Protection Regulation for clinical studies ……… 9
- Part II. General aspects related to (electronic) informed consent ………………………….. 31

Questions for ethics committees …………………………………………………………………... 43

- Introductory questions ……………………………………………………………………... 43
- Part I. Compliance with the General Data Protection Regulation for clinical studies …….. 45
- Part II. General aspects related to (electronic) informed consent …………………………. 58

Questions for investigators (physicians) ………….……………………………………………….. 67

- Introductory questions ……………………………………………………………………... 67
- Part I. Compliance with the General Data Protection Regulation for clinical studies …….. 69
- Part II. General aspects related to (electronic) informed consent …………………………. 91

**Start of Block: Introduction**

Q1
**Investigating challenges related to data protection and informed consent in clinical research during the COVID-19 pandemic**

 ***Welcome to Our Survey***
 
*This survey is conducted in the context of research at KU Leuven and can be used to inform other projects, such as the IMI-2 consortium Corona Accelerated R&D in Europe (CARE).*

 The **COVID-19 pandemic** has brought global disruption to health, society, and economy, including the conduct of clinical research. In the European Union, the legal and ethical framework for clinical research is very complex and highly divergent. **Many challenges** exist in relation to **the interplay of the various applicable rules**. In particular, with respect to the compliance with the EU General Data Protection Regulation (GDPR). In addition, ethical challenges regarding taking part in a clinical study were raised as the paper-based informed consent process has been affected by the COVID-19 pandemic.

 The **aim of this survey** is to gain insights into the experience of clinical research stakeholders on key challenges and related solutions prior to and during the COVID-19 pandemic. The results of this survey will be used **solely for scientific purposes** and will be published in academic journals. They will support researchers in being compliant with the GDPR and in understanding the legal and ethical framework in drug development during the COVID-19 pandemic. For example, the results will inform the CARE consortium. 
 
The **survey consists of two parts**: 
**Part 1.** Compliance with the General Data Protection Regulation for clinical studies
**Part 2.**General aspects related to (electronic) informed consent

 **At the end of Part 1**, you will be asked if you want to participate in a **follow-up interview** that will explore in more depth the issues **related to data protection**. For this, your **contact details** will be requested. During the interview, we would go over your answers in the survey and discuss some follow-up questions. Your additional insights will help us to further refine recommendations.

 The survey will take **approximately 25-30 minutes** to complete. 
 
**You can interrupt the survey and resume at a later time** if you use the same device and browser. 

 Participation in the survey is **voluntary**. You can withdraw at any time, without any consequences.

 In the scope of this survey, KU Leuven will process your data as data controller for the purposes that were presented to you. The legal basis for processing your personal data is public interest (Article 6(1)(f) of Regulation (EU) 2016/679).

 All research data will be stored for a period of 10 years. Your personal data may be stored for an additional period of time, if necessary, for the purposes of this study, or for further research on the topic.

 The study was approved by the Ethics Committee Research UZ/KU Leuven (S65106).

 For more information, please refer to [KU Leuven privacy policy](https://admin.kuleuven.be/privacy/en) and [Qualtrics privacy policy](https://www.qualtrics.com/support/survey-platform/getting-started/data-protection-privacy/).

 If you have any further questions regarding this study, please do not hesitate to contact:
**Teodora Lalova**, PhD researcher KU Leuven ([teodora.lalova@kuleuven.be](mailto:teodora.lalova@kuleuven.be)  or +32 2 774 16 24)
 **Evelien De Sutter**, PhD researcher KU Leuven ([evelien.desutter@kuleuven.be](mailto:evelien.desutter@kuleuven.be) or +32 16 37 98 82) 

 Thank you for participating in this study!

 Do you agree with the terms presented to you above? You must select "Yes" in order to complete the survey.

- Yes (this option proceeds with the survey) (1)
- No (this option automatically terminates the survey) (2)

| Page Break |  |
| --- | --- |

**End of Block: Introduction**

**Start of Block: Stakeholder groups**

Q2 **Introductory questions**

| 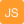 |
| --- |

Q3 Which stakeholder group best characterises you?  *
*(Select only one)*

- I am a data protection officer or a member of a legal team (and I am NOT a member of an ethics committee) (1)
- I am a member of an ethics committee (2)
- I am an investigator/physician (and I am NOT a member of an ethics committee) (3)
- None of the listed stakeholder groups (5)

**End of Block: Stakeholder groups**

**Start of Block: Introductory questions DPO**

Q4 Are you a member of the IMI-2 consortium Corona Accelerated R&D in Europe (CARE)? *

- Yes (1)
- No (2)

Q5 Where do you work?&nbsp*


(*In case you work for more than one employer, please select the institution from the perspective of which you prefer to answer this survey)*

- An academic sponsor of clinical trials (1)
- A pharmaceutical company (2)
- A biobank (3)
- A clinical research organisation (CRO) (4)
- A research institute (5)
- A hospital (6)
- Other *(please specify)* (7) ________________________________________________

Q6 Where is your organisation based? *

▼ Austria (1) ... United Kingdom (28)

Q7 In which country is your organisation involved in clinical studies? *

- Only in the country where it is based (national studies) (1)
- In several countries (international studies, *please specify which countries)* (2) ________________________________________________

Q8 Please select the answer that is applicable to you - I was/am involved in:*
*(Multiple answers possible)*

- **COVID-19 studies:** clinical studies that investigate a COVID-19 medicine, diagnostic product or device, and/or vaccine (1)
- **Non-COVID-19 studies**: clinical studies that investigate a medicine, diagnostic product or device, and/or vaccine for other medical conditions (e.g. cancer) (2)

Q9 According to applicable legislation, there are different types of clinical studies. Below, we list several types of clinical studies (you can find the definitions by hovering over their names).

 **Please select the types of studies in which you are involved most often.***
 *(Multiple answers possible)*

- Interventional clinical trials (1)
- Non-interventional clinical trials (2)
- Clinical investigation of a medical device (3)
- Clinical performance study of an in vitro diagnostic medical device (5)

| 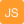 |
| --- |

Q10 Have you conducted a registry-based trial (i.e. an investigation of a research question which uses the infrastructure of a new or an existing registry for patient recruitment and/or data collection)? *

- Yes (1)
- No (2)

| Page Break |  |
| --- | --- |

**End of Block: Introductory questions DPO**

**Start of Block: Part I. DPO**

Q11 **Part I. Compliance with the General Data Protection Regulation for clinical studies**

Q12 **Topic 1. PRIMARY use of personal data for research purposes**

 *In this survey,* ***"primary use"****of personal data for research means the use of data* ***directly collected*** *and used for the purposes of clinical studies. For example, primary use purposes would include projects that were named at the time of data collection.*

Q13


**How many legal bases do you rely on in the scope of one clinical study?**

 *During the* ***lifecycle of a clinical study****, there are* ***two main categories of processing operations****:*

*- Processing operations purely related to RESEARCH purposes,*

*- Processing operations related to SAFETY purposes (for instance, notification of adverse events to competent authorities)*
  
*A* ***valid legal basis*** *is needed to process personal data. Pursuant to GDPR, there are* ***six legal bases****, specified in Article 6(1). By hovering over this text, you can see a summary of these legal bases.*

- One legal basis (for both processing operations related to research and related to safety purposes) (1)
- Two legal bases (one for processing operations related to research, and one for operations related to safety purposes) (2)

| 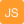 |
| --- |

Q14 Do you think that the processing of special categories of personal data (such as data concerning health, race, ethnicity, genetics) should be based on a combination of a legal basis under
**Article 6(1)** and a special condition under
**Article 9(2)**GDPR? 
 You can find a summary of Articles 6(1) and 9(2) by hovering over the text in bold above.

- Yes, it should be a combination (I apply Article 6(1) in conjunction with Article 9(2)). (1)
- No, it should not be a combination (I apply only Article 9(2)). (28)

*Display This Question:*

*If Q14 = Yes, it should be a combination (I apply Article 6(1) in conjunction with Article 9(2)).*

*And Q13 = Two legal bases (one for processing operations related to research, and one for operations related to safety purposes)*

Q15 **For primary use of personal data for RESEARCH purposes**, please indicate **how frequently** your organisation relies on each of the six legal bases available under **Article 6(1) GDPR**. *(By hovering over the legal bases, you can find a short explanation and a reference to the law)*

|  | Never (1) | Sometimes (2) | About half the time (3) | Most of the time (4) | Always (5) |
| --- | --- | --- | --- | --- | --- |
| Consent (1) |  |  |  |  |  |
| Contract (2) |  |  |  |  |  |
| Legal obligation (3) |  |  |  |  |  |
| Vital interests (4) |  |  |  |  |  |
| Public interest (5) |  |  |  |  |  |
| Legitimate interests (7) |  |  |  |  |  |

*Display This Question:*

*If Q14 = Yes, it should be a combination (I apply Article 6(1) in conjunction with Article 9(2)).*

*And Q13 = Two legal bases (one for processing operations related to research, and one for operations related to safety purposes)*

Q16 **For primary use of special categories of personal data (**such as data concerning health - medical history, results of examinations and treatments etc**) for RESEARCH purposes**, please indicate **how frequently** your organisation relies on each of the listed below special conditions available under **Article 9(2) GDPR**. *(By hovering over the conditions, you can find a short explanation and a reference to the law)*

|  | Never (1) | Sometimes (2) | About half the time (3) | Most of the time (4) | Always (5) |
| --- | --- | --- | --- | --- | --- |
| Explicit consent (1) |  |  |  |  |  |
| Vital interests (2) |  |  |  |  |  |
| Personal data manifestly made public (3) |  |  |  |  |  |
| Substantial public interest (4) |  |  |  |  |  |
| Preventive or occupational medicine (5) |  |  |  |  |  |
| Public interest in the area of public health (6) |  |  |  |  |  |
| Scientific research (7) |  |  |  |  |  |

*Display This Question:*

*If Q14 = Yes, it should be a combination (I apply Article 6(1) in conjunction with Article 9(2)).*

*And Q13 = Two legal bases (one for processing operations related to research, and one for operations related to safety purposes)*

Q17 If you **indicated that your organisation relies on some legal bases/special conditions "Most of the time" and "Always"** for the primary use of personal data for RESEARCH purposes, please answer:
 
**During the COVID-19 pandemic**, did your organisation use different legal bases/special conditions? 

- Yes, during the pandemic we relied on different legal bases and special conditions. *(Please specify why and provide examples)* (1) ________________________________________________
- No (2)

*Display This Question:*

*If Q14 = No, it should not be a combination (I apply only Article 9(2)).*

*And Q13 = Two legal bases (one for processing operations related to research, and one for operations related to safety purposes)*

Q18
For primary use of special categories of personal data (such as data concerning health - medical history, results of examinations and treatments etc) for RESEARCH purposes, please indicate how frequently your organisation relies on each of the listed below special conditions available under Article 9(2) GDPR. *(By hovering over the conditions, you can find a short explanation and a reference to the law)*
 
 

|  | Never (1) | Sometimes (2) | About half the time (3) | Most of the time (4) | Always (5) |
| --- | --- | --- | --- | --- | --- |
| Explicit consent (1) |  |  |  |  |  |
| Vital interests (2) |  |  |  |  |  |
| Personal data manifestly made public (3) |  |  |  |  |  |
| Substantial public interest (4) |  |  |  |  |  |
| Preventive or occupational medicine (5) |  |  |  |  |  |
| Public interest in the area of public health (6) |  |  |  |  |  |
| Scientific research (7) |  |  |  |  |  |

*Display This Question:*

*If Q14 = No, it should not be a combination (I apply only Article 9(2)).*

*And Q13 = Two legal bases (one for processing operations related to research, and one for operations related to safety purposes)*

Q19
If you **indicated that your organisation relies on some special conditions "Most of the time" and "Always"** for the use of personal data for RESEARCH purposes, please answer: 
 
**During the COVID-19 pandemic**, did this change?

- Yes, during the pandemic, my organisation relied on different special conditions. *(Please specify why and provide examples)* (1) ________________________________________________
- No (2)

*Display This Question:*

*If Q13 = One legal basis (for both processing operations related to research and related to safety purposes)*

*And Q14 = Yes, it should be a combination (I apply Article 6(1) in conjunction with Article 9(2)).*

Q20 **For primary use of personal data for RESEARCH and SAFETY purposes**, please indicate **how frequently** your organisation relies on each of the six legal bases available under **Article 6(1) GDPR**. *(By hovering over the legal bases, you can find a short explanation and a reference to the law)*

|  | Never (1) | Sometimes (2) | About half the time (3) | Most of the time (4) | Always (5) |
| --- | --- | --- | --- | --- | --- |
| Consent (1) |  |  |  |  |  |
| Contract (2) |  |  |  |  |  |
| Legal obligation (3) |  |  |  |  |  |
| Vital interests (4) |  |  |  |  |  |
| Public interest (5) |  |  |  |  |  |
| Legitimate interests (6) |  |  |  |  |  |

*Display This Question:*

*If Q13 = One legal basis (for both processing operations related to research and related to safety purposes)*

*And Q14 = Yes, it should be a combination (I apply Article 6(1) in conjunction with Article 9(2)).*

Q21 **For the primary use**of special categories of personal data (such as data concerning health - medical history, results of examinations and treatments etc) **for RESEARCH and SAFETY purposes**, please indicate **how frequently** your organisation relies on each of the listed below special conditions available under **Article 9(2) GDPR**. *(By hovering over the conditions, you can find a short explanation and a reference to the law)*

|  | Never (1) | Sometimes (2) | About half the time (3) | Most of the time (4) | Always (5) |
| --- | --- | --- | --- | --- | --- |
| Explicit consent (1) |  |  |  |  |  |
| Vital interests (2) |  |  |  |  |  |
| Personal data manifestly made public (3) |  |  |  |  |  |
| Substantial public interest (4) |  |  |  |  |  |
| Preventive or occupational medicine (5) |  |  |  |  |  |
| Public interest in the area of public health (6) |  |  |  |  |  |
| Scientific research (7) |  |  |  |  |  |

*Display This Question:*

*If Q13 = One legal basis (for both processing operations related to research and related to safety purposes)*

*And Q14 = Yes, it should be a combination (I apply Article 6(1) in conjunction with Article 9(2)).*

Q22
If you **indicated that your organisation relies on some legal bases/special conditions "Most of the time" and "Always"** for the use of personal data for RESEARCH and SAFETY purposes, please answer: 
 
**During the COVID-19 pandemic**, did this change?

- Yes, during the pandemic, my organisation relied on different legal bases/special conditions. *(Please specify why and provide examples)* (1) ________________________________________________
- No (2)

*Display This Question:*

*If Q13 = One legal basis (for both processing operations related to research and related to safety purposes)*

*And Q14 = No, it should not be a combination (I apply only Article 9(2)).*

Q23 For primary use of special categories of personal data (such as data concerning health - medical history, results of examinations and treatments etc) for RESEARCH and SAFETY purposes, please indicate how frequently your organisation relies on each of the listed below special conditions available under Article 9(2) GDPR.*(By hovering over the conditions, you can find a short explanation and a reference to the law)*

|  | Never (1) | Sometimes (2) | About half the time (3) | Most of the time (4) | Always (5) |
| --- | --- | --- | --- | --- | --- |
| Explicit consent (1) |  |  |  |  |  |
| Vital interests (2) |  |  |  |  |  |
| Personal data manifestly made public (3) |  |  |  |  |  |
| Substantial public interest (4) |  |  |  |  |  |
| Preventive or occupational medicine (5) |  |  |  |  |  |
| Public interest in the area of public health (6) |  |  |  |  |  |
| Scientific research (7) |  |  |  |  |  |

*Display This Question:*

*If Q13 = One legal basis (for both processing operations related to research and related to safety purposes)*

*And Q14 = No, it should not be a combination (I apply only Article 9(2)).*

Q24
If you **indicated that your organisation relies on some special conditions "Most of the time" and "Always"** for the use of personal data for RESEARCH and SAFETY purposes, please answer: 
  During the COVID-19 pandemic, did this change?

- Yes, during the pandemic, my organisation relied on different special conditions. *(Please specify why and provide examples)* (1) ________________________________________________
- No (2)

*Display This Question:*

*If Q13 = Two legal bases (one for processing operations related to research, and one for operations related to safety purposes)*

*And Q14 = Yes, it should be a combination (I apply Article 6(1) in conjunction with Article 9(2)).*

*Or Q13 = Two legal bases (one for processing operations related to research, and one for operations related to safety purposes)*

*And Q14 = No, it should not be a combination (I apply only Article 9(2)).*

*Or Q13 = One legal basis (for both processing operations related to research and related to safety purposes)*

*And Q14 = Yes, it should be a combination (I apply Article 6(1) in conjunction with Article 9(2)).*

*Or Q13 = One legal basis (for both processing operations related to research and related to safety purposes)*

*And Q14 = No, it should not be a combination (I apply only Article 9(2)).*

Q25 **If in the previous questions, you indicated "Most of the time" or "Always"** to any of the legal bases/special conditions listed below, please provide **a reference to the national law**, which implements the relevant provision. 
 
If your organisation conducts studies in several countries, please provide reference to all national laws that you are aware of.

- **Legal obligation:** processing is necessary for compliance with a legal obligation (Article 6(1)(c)) (1) ________________________________________________
- **Public interest:** processing is necessary for the performance of a task carried out in the public interest (Article 6(1)(e)) (2) ________________________________________________
- **Substantial public interest:** processing is necessary for reasons of substantial public interest (Article 9(2)(g) (3) ________________________________________________
- **Public interest in the area of public health:** processing is necessary for public interest in the area of public health (Article 9(2)(i)) (4) ________________________________________________
- **Scientific research:** processing is necessary for archiving purposes in the public interest, scientific or historical research purposes or statistical purposes (Article 9(2)(j)) (5) ________________________________________________
- I do not know (6)
- Not applicable to me (7)

*Display This Question:*

*If Q7 = In several countries (international studies, <em>please specify which countries)</em>*

Q26 When your organisation conducts **one clinical study in several countries**, **how often do they rely on the same legal basis** for the use of personal data in each country?

|  | Never (1) | Sometimes (2) | About half the time (3) | Most of the time (4) | Always (5) |
| --- | --- | --- | --- | --- | --- |
| For **one clinical study in several countries**, my organisation relies on the **same legal basis**: (7) |  |  |  |  |  |

*Display This Question:*

*If Q26 = Never*

*Or Q26 = Sometimes*

Q27 **Why** does your organisation rely on a **different legal basis** in each country?

________________________________________________________________

________________________________________________________________

________________________________________________________________

________________________________________________________________

________________________________________________________________

| Page Break |  |
| --- | --- |

Q28 **Topic 2. SECONDARY use of personal data for research purposes**
 
*In this survey,* ***"secondary use"*** *of personal data for research means the* ***re-use (further processing)*** *of personal data that was* ***initially collected and used for another purpose****. For example, when health data which was originally collected in the scope of care or in a previous clinical study, is used for the conduct of another study, this would be considered secondary use. 

 Personal data cannot be re-used if that would be incompatible with the initial purposes for collection and use.*

| 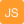 |
| --- |

Q29 **When you re-use personal data from a previous project, how often does your organisation rely on the presumption of compatibility for research (Article 5(1)(b) GDPR)?**

 *The* ***presumption of compatibility*** *means that the re-use of personal data for scientific research purposes is considered compatible with the initial purposes, provided that appropriate safeguards are in place.*

|  | Never (1) | Sometimes (2) | About half the time (3) | Most of the time (4) | Always (5) |
| --- | --- | --- | --- | --- | --- |
| For the re-use of personal data, we rely on the presumption of compatibility: (1) |  |  |  |  |  |

*Display This Question:*

*If Q29 = Most of the time*

*Or Q29 = Always*

*Or Q29 = For the re-use of personal data, we rely on the presumption of compatibility: [ Most of the time ]*

*Or Q29 = For the re-use of personal data, we rely on the presumption of compatibility: [ Always ]*

*Or Q29 = About half the time*

*Or Q29 = Sometimes*

Q30 **What appropriate safeguards** does your organisation put in place when relying on the presumption of compatibility?
*(Multiple answers possible)*

- **Inform** study participants about the new research project within a reasonable period of time before the project is implemented. (4)
- **Pseudonymise** the personal data of study participants (5)
- **Obtain the informed consent** of the study participants*. (Informed consent is understood here as the ethical requirement for participation in research, and not as consent as a legal basis under the GDPR.)* (6)
- Other *(please give examples)* (7) ________________________________________________

*Display This Question:*

*If Q29 = Most of the time*

*Or Q29 = Always*

*Or Q29 = For the re-use of personal data, we rely on the presumption of compatibility: [ Most of the time ]*

*Or Q29 = For the re-use of personal data, we rely on the presumption of compatibility: [ Always ]*

Q31 If you are aware of a national law/policy that provides examples of appropriate safeguards, please share the reference:

________________________________________________________________

*Display This Question:*

*If Q29 = Most of the time*

*Or Q29 = Always*

*Or Q29 = For the re-use of personal data, we rely on the presumption of compatibility: [ Most of the time ]*

*Or Q29 = For the re-use of personal data, we rely on the presumption of compatibility: [ Always ]*

Q32 Which statement do you agree with?

- When I re-use personal data for research purposes, **I can rely on the same legal basis** as for the primary use (1)
- **I need to have a new legal basis** in order to re-use personal data for research purposes (2)
- **Both options** listed above are valid (3)
- **I do not think that a legal basis is required at all** for the re-use of personal data for research (4)
- No opinion (5)

Q33
**How often does your organisation conduct the compatibility assessment when re-using personal data for research?**

 ***Compatibility assessment*** *Personal data can be re-used for another purpose (e.g. a new study) after checking that the new purpose is compatible with the original purpose for which the data was collected. This check is known as the compatibility assessment (Article 6(4)), for which the GDPR established five elements that should be considered. (You can see a summary of these elements by hovering over the text above)*

|  | Never (1) | Sometimes (2) | About half the time (3) | Most of the time (4) | Always (5) |
| --- | --- | --- | --- | --- | --- |
| We conduct the compatibility assessment: (8) |  |  |  |  |  |

*Display This Question:*

*If Q33 = Most of the time*

*Or Q33 = Always*

*Or Q33 = We conduct the compatibility assessment: [ Most of the time ]*

*Or Q33 = We conduct the compatibility assessment: [ Always ]*

*Or Q33 = Sometimes*

*Or Q33 = About half the time*

Q34 Please rank **from most (1) to least important (5)**the elements of the compatibility assessment. 


You can do so by dragging and dropping each item.

______ **Any link between the purposes** for which the personal data have been collected and the purposes of the intended further processing. (1)

______ **The context** in which the personal data have been collected (what is the relationship between your organisation and the individual). (2)

______ **The nature of the personal data**, in particular whether special categories of personal data are processed (such as data concerning health). (3)

______ **The possible consequences** of the intended further processing for data subjects (how it will impact the individuals). (4)

______ The existence of **appropriate safeguards** (for instance encryption or pseudonymisation). (5)

| 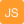 |
| --- |

Q35 **During the COVID-19 pandemic**, did anything change in the way that your organisation processes personal data for secondary use?

- Yes *(please specify how)* (1) ________________________________________________
- No (2)

| Page Break |  |
| --- | --- |

Q36 **Topic 3. Transparency**

Q37 When your organisation **collects personal data for primary use** for research, **do they clearly inform** study participants **about the categories of data** (e.g., age, sex, drug test results, etc) that are being collected?

- Yes (1)
- No (2)

Q38 **Prior to the COVID-19 pandemic**, what were the **biggest challenges** that your organisation experienced when providing GDPR-related information to study participants?

________________________________________________________________

| 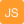 |
| --- |

Q39 **During the COVID-19 pandemic**, are the biggest c**hallenges still the same** as the ones you listed above, or different?

- The same (1)
- Different *(please specify)* (2) ________________________________________________

| Page Break |  |
| --- | --- |

Q40 **Topic 4. Communication with ethics committees**

Q41
Do you consider that the GDPR establishes a special role for ethics committees when it comes to the way that research organisations should process personal data for scientific research?

- Yes *(please specify)* (1) ________________________________________________
- No (2)

*Display This Question:*

*If Q41 = No*

Q42 Do you think that ethics committees should have such a special role?

- Yes *(please specify why)* (1) ________________________________________________
- No *(please specify why)* (2) ________________________________________________

Q43 Does your organisation receive comments from ethics committees in relation to GDPR-related text in study protocols? For instance, asking for the deletion or inclusion of specific text.

- Yes *(please provide examples)* (1) ________________________________________________
- No (2)
- I do not know (3)

*Display This Question:*

*If Q43 = Yes <em>(please provide examples)</em>*

Q44 Do you think that if you follow some of these ethics committees' comments, your study may become non-compliant with the GDPR?

- Yes *(please specify)* (1) ________________________________________________
- No (2)
- I do not know (3)

Q45 Do ethics committees sometimes insist on the use of a specific legal basis under GDPR **for the primary use** of personal data for research?

- Yes *(please provide examples)* (1) ________________________________________________
- No (2)
- I do not know (3)

Q46 Do ethics committees sometimes insist on the use of a specific legal basis under GDPR **for the secondary use** of personal data for research?

- Yes *(please provide examples)* (1) ________________________________________________
- No (2)
- I do not know (3)

| 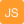 |
| --- |

Q47 Do ethics committees sometimes insist on the use of a **specific technique for anonymisation or pseudonymisation** of personal data?

- Yes *(please specify)* (1) ________________________________________________
- No (2)
- I do not know (3)

| Page Break |  |
| --- | --- |

Q48 **Topic 5. Challenges prior to and during the COVID-19 pandemic**

Q49 **Prior to the pandemic**, in relation to which of the **topics** below did your organisation experience **challenges**?
 
You can also specify additional challenges that were not listed (box "Other").

|  | Never (1) | Sometimes (2) | About half the time (3) | Most of the time (4) | Always (5) |
| --- | --- | --- | --- | --- | --- |
| Choice of legal basis for PRIMARY use of personal data (1) |  |  |  |  |  |
| SECONDARY use of personal data (2) |  |  |  |  |  |
| Providing information to study participants (3) |  |  |  |  |  |
| Communication with ethics committees (4) |  |  |  |  |  |
| Lack of legal harmonisation in case of multi-site studies in different countries (5) |  |  |  |  |  |
| Other *(please specify)* (6) |  |  |  |  |  |

| 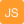 |
| --- |

Q50 **During the pandemic**, in relation to which of the **topics** below did your organisation experience **challenges**?
 
You can also specify additional challenges that were not listed (box "Other").

|  | Never (1) | Sometimes (2) | About half the time (3) | Most of the time (4) | Always (5) |
| --- | --- | --- | --- | --- | --- |
| Choice of legal basis for PRIMARY use of personal data (1) |  |  |  |  |  |
| SECONDARY use of personal data (2) |  |  |  |  |  |
| Providing information to study participants (3) |  |  |  |  |  |
| Communication with ethics committees (4) |  |  |  |  |  |
| Lack of legal harmonisation in case of multi-site studies in different countries (5) |  |  |  |  |  |
| Other *(please specify)* (6) |  |  |  |  |  |

| Page Break |  |
| --- | --- |

Q51 **Closing questions to Part I. Compliance with the General Data Protection Regulation for clinical studies**

Q52 Do you want to mention an additional point of concern regarding **data protection**, not covered in this survey?

________________________________________________________________

| 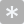 |
| --- |

Q53 Please share your email address, if you would like to participate in a follow-up interview that will explore in more depth the **issues related to data protection**.

________________________________________________________________

| 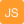 |
| --- |

Q54 **You reached the end of Part I. The next set of questions deals with eConsent.**

**End of Block: Part I. DPO**

**Start of Block: Part II. DPO**

Q55 **Part II. General aspects related to (electronic) informed consent**

Q56
**Topic 1. General question**

| 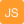 |
| --- |

Q57 What would be the ideal means of communication, **regardless of the COVID-19 pandemic**, to inform research participants about the objectives and conduct of a clinical study (e.g. study procedures, risks, benefits...)?

________________________________________________________________

________________________________________________________________

________________________________________________________________

________________________________________________________________

________________________________________________________________

| Page Break |  |
| --- | --- |

Q58
**Topic 2. Electronic informed consent**

Q59 Does your organisation have experience with **informing research participants and obtaining their informed consent** via electronic means?

- Yes (1)
- No (2)
- I do not know (3)

*Display This Question:*

*If Q59 = Yes*

Q60 For which clinical study(ies) does your organisation use electronic means to inform research participants and obtain their consent? 
*(You can find more information about each study type by hovering over the answer options - Multiple answers possible)*

- Interventional clinical trials (1)
- Non-interventional clinical trials (2)
- Clinical investigation of a medical device (3)
- Clinical performance study of an in vitro diagnostic medical device (4)

*Display This Question:*

*If Q59 = Yes*

Q61 Which electronic mean(s) does your organisation use to inform research participants and obtain their consent? 
*(Multiple answers possible)*

- Phone (1)
- Tablet (2)
- Computer (3)
- Other *(please specify)* (4) ________________________________________________

*Display This Question:*

*If Q7 = Only in the country where it is based (national studies)*

Q62 Does national law or policy of the country in which your organisation operates, provide a **definition** of electronic informed consent?

- Yes *(please specify)* (1) ________________________________________________
- No (2)
- I do not know (3)

*Display This Question:*

*If Q7 = In several countries (international studies, <em>please specify which countries)</em>*

Q63
Does national law or policy of at least one of the countries in which your organisation operates, provide a definition of electronic informed consent?

- Yes *(please specify which country/countries and definition(s))* (1) ________________________________________________
- No (2)
- I do not know (3)

*Display This Question:*

*If Q62 = No*

*Or Q62 = I do not know*

*Or Q63 = No*

*Or Q63 = I do not know*

Q64 What do you understand by electronic informed consent?

________________________________________________________________

________________________________________________________________

________________________________________________________________

________________________________________________________________

________________________________________________________________

Q65 Which **functionalities** should be part of an electronic informed consent platform (which is a platform enabling research participants to give and manage their electronic informed consent)? 
*(Multiple answers possible)*

- Providing research study information in an interactive and dynamic way to (potential) research subjects (1)
- Obtaining and documenting the signature of the research subjects (2)
- The return of research results to the research subjects (3)
- The possibility to reconsent research subjects (4)
- Other *(please specify)* (5) ________________________________________________

*Display This Question:*

*If Q7 = Only in the country where it is based (national studies)*

Q66
Is it legally allowed in the country where your organisation operates to **provide study-related information** to research participants via electronic means, before obtaining their informed consent?

- Yes (1)
- Only under certain conditions *(please specify)* (3) ________________________________________________
- No (4)
- I do not know (5)

*Display This Question:*

*If Q7 = In several countries (international studies, <em>please specify which countries)</em>*

Q67
Is it legally allowed in at least one of the countries where your organisation operates to provide study-related information to research participants via electronic means, before obtaining their informed consent?

- Yes *(please specify which country(ies))* (1) ________________________________________________
- Only under certain conditions *(please specify which country(ies) and conditions)* (2) ________________________________________________
- No (3)
- I do not know (4)

*Display This Question:*

*If Q7 = Only in the country where it is based (national studies)*

Q68 Is it legally allowed in the country where your organisation operates to obtain research participants' informed **consent** via electronic means?

- Yes (1)
- Only under certain conditions *(please specify)* (2) ________________________________________________
- No (3)
- I do not know (4)

*Display This Question:*

*If Q7 = In several countries (international studies, <em>please specify which countries)</em>*

Q69
Is it legally allowed in at least one of the countries where your organisation operates to obtain research participants' informed consent via electronic means?

- Yes *(please specify which country(ies))* (1) ________________________________________________
- Only under certain conditions  *(please specify which country(ies) and conditions)* (2) ________________________________________________
- No (3)
- I do not know (4)

*Display This Question:*

*If Q68 = Yes*

*Or Q68 = Only under certain conditions<em> (please specify)</em>*

*Or Q69 = Yes<em> (please specify which country(ies))</em>*

*Or Q69 = Only under certain conditions <em> (please specify which country(ies) and conditions)</em>*

Q70 Which **signature(s)** is/are legally allowed to obtain the research participants' informed consent?
*(You can find the definitions of the different signatures by hovering over the answer options - Multiple answers possible)*

- Simple or basic electronic signature (1)
- Advanced electronic signature (2)
- Qualified advanced electronic signature (3)
- I do not know (4)
- Other *(please specify)* (5) ________________________________________________

| 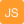 |
| --- |

Q71 Which laws or regulations regulate (i.e. allow or prohibit) the use of electronic informed consent?

________________________________________________________________

________________________________________________________________

________________________________________________________________

________________________________________________________________

________________________________________________________________

| Page Break |  |
| --- | --- |

Q72 **Topic 3. Informed consent during the COVID-19 pandemic**

Q73 Alternative methods were recommended, for example by the European Medicines Agency, to **re-consent for already included research participants**, e.g. due to protocol changes. These methods include:
 
**Method 1**: Obtaining oral consent (e.g. via phone or video-calls), supplemented with e-mail confirmation.
 
**Method 2**: Obtaining oral consent (e.g. via phone or video-calls). An appropriately signed and dated informed consent form should be obtained from the research participant as soon as possible.
 
**Method 3**: Using validated electronic systems, e.g. electronic informed consent.
 
Which method(s) did your organisation employ during the COVID-19 pandemic? 
*(Multiple answers possible)*

- Method 1 (1)
- Method 2 (2)
- Method 3 (3)
- Other method *(please specify)* (4) ________________________________________________
- Not applicable (5)

Q74 How useful are the alternative methods to re-consent for **already included research participants**? *(You can find more information about each method by hovering over their names)*

|  | Not useful at all (35) | Slightly useful (36) | Moderately useful (38) | Extremely useful (40) |
| --- | --- | --- | --- | --- |
| **Method 1** (1) |  |  |  |  |
| **Method 2** (2) |  |  |  |  |
| **Method 3** (3) |  |  |  |  |
| **Other *(if any)*** (5) |  |  |  |  |

*Display This Question:*

*If Q74 = Not useful at all*

*Or Q74 = Slightly useful*

Q75 Please specify why the recommended method(s) is/are not useful at all or slightly useful

________________________________________________________________

________________________________________________________________

________________________________________________________________

________________________________________________________________

________________________________________________________________

*Display This Question:*

*If Q74 = Moderately useful*

*Or Q74 = Extremely useful*

Q76 Please specify why the recommended method(s) is/are moderately or extremely useful

________________________________________________________________

________________________________________________________________

________________________________________________________________

________________________________________________________________

________________________________________________________________

Q77
Specific methods were recommended, for example by the European Medicines Agency, when obtaining informed consent of **COVID-19 patients**. These methods include:
 
Method 1: If written consent by the research participant is not possible, consent could be given orally by the research participant in the presence of an impartial witness. In such cases, the witness is required to sign and date the informed consent form and the investigator is expected to record how the impartial witness was selected.
 
Method 2: The research participant and the person obtaining consent sign and date separate informed consent forms (i.e. one informed consent form signed and dated by the participant and another consent form signed and dated by the researcher). An appropriately signed and dated informed consent should be obtained from the research participant later, as soon as possible.
 
Method 3: Using validated electronic systems, e.g. electronic informed consent.
 
Which method(s) did your organisation employ during the COVID-19 pandemic? 
*(Multiple answers possible)*

- Method 1 (1)
- Method 2 (2)
- Method 3 (3)
- Other method *(please specify)* (4) ________________________________________________
- Not applicable (5)

Q78 How useful are the alternative methods for obtaining informed consent of **COVID-19 patients**?
*(You can find more information about each method by hovering over their names)*

|  | Not useful at all (1) | Slightly useful (2) | Moderately useful (3) | Extremely useful (4) |
| --- | --- | --- | --- | --- |
| **Method 1 (8)** |  |  |  |  |
| **Method 2** (9) |  |  |  |  |
| **Method 3** (10) |  |  |  |  |
| **Other *(if any)*** (11) |  |  |  |  |

*Display This Question:*

*If Q78 = Not useful at all*

*Or Q78 = Slightly useful*

Q79 Please specify why the recommended method(s) is/are not useful at all or slightly useful

________________________________________________________________

________________________________________________________________

________________________________________________________________

________________________________________________________________

________________________________________________________________

*Display This Question:*

*If Q78 = Moderately useful*

*Or Q78 = Extremely useful*

Q80 Please specify why the recommended method(s) is/are moderately or extremely useful

________________________________________________________________

________________________________________________________________

________________________________________________________________

________________________________________________________________

________________________________________________________________

Q81 **Prior to the pandemic**, what were the biggest challenges that you experienced when providing research information to participants and obtaining consent?

________________________________________________________________

| 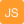 |
| --- |

Q82 **During the pandemic**, are these challenges the same, or different?

- The same (2)
- Different *(please specify why)* (3) ________________________________________________

| Page Break |  |
| --- | --- |

**End of Block: Part II. DPO**

**Start of Block: Closing question part II**

Q300 Closing question to Part II. General aspects related to (electronic) informed consent

| 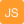 |
| --- |

Q301 Do you want to mention an additional point of concern regarding eConsent, not covered in this survey?

________________________________________________________________

**End of Block: Closing question part II**

**Start of Block: Introductory questions Ethics committees**

Q83 Are you a member of the IMI-2 consortium Corona Accelerated R&D in Europe (CARE)? *

- Yes (1)
- No (2)

Q84 What is your profession? *

- I am a physician (1)
- I am a lawyer (2)
- I am a patient representative (3)
- I am a study nurse (4)
- I am a statistician (5)
- I am a philosopher/priest (6)
- Other *(please specify)* (7) ________________________________________________

Q85 Where is your organisation based? *

▼ Austria (1) ... United Kingdom (28)

Q86 The ethical committee of which I am a member: *

- Is a national ethics committee (1)
- Is a local or regional ethics committee (2)

Q87 What types of clinical studies does the ethics committee of which you are a member assess? * 
*(Multiple answers possible)*

- Clinical studies that are conducted only in the country where the ethics committee is based (1)
- Clinical studies that are conducted in several EU countries (2)

| 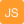 |
| --- |

Q88 According to applicable legislation, there are different types of clinical studies. Below, we list several types of clinical studies (you can find the definitions by hovering over their names).
 

**Please select the types of studies in which you are involved most often. ***
*(Multiple answers possible)*

- Interventional clinical trials (1)
- Non-interventional clinical trials (2)
- Clinical investigation of a medical device (3)
- Clinical performance study of an in vitro diagnostic medical device (5)

**End of Block: Introductory questions Ethics committees**

**Start of Block: Part I. Ethics committees**

Q89 Part I. Compliance with the General Data Protection Regulation for clinical studies

Q90 **Topic 1. General questions**

Q91 Has your ethics committee been formally trained in data protection?

- Yes (1)
- No (2)

Q92 Does an individual member of your ethics committee have training in data protection?
*(Multiple answers possible)*

- Yes, I have such a training (1)
- Yes, someone other than myself has such a training (2)
- None of the members of my ethics committee has been trained in data protection (4)

Q93 How could knowledge about data protection among ethics committees be enhanced?

________________________________________________________________

Q94 Is there a national law or a local and/or institutional policy in your country of residence, which establishes a special role for ethics committees in data protection?

- Yes *(please specify and provide a link or a reference, if possible)* (1) ________________________________________________
- No (2)

| 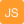 |
| --- |

Q95
**In your view, does the ethics committee process personal data in the scope of its activities (for instance, when it is notified for adverse events)?**
  
***"Personal data"*** *is any information about a particular natural person that allows, or could allow identifying the person. Examples of personal data include name and surname, home address. There are also****special categories of personal data****, such as patients' health and genetic data (e.g. medical history, results of examinations and treatments, etc). Special categories of data are considered to be more sensitive, and the GDPR gives them extra protection.* 
 
***"Processing" of personal data*** *covers a wide range of operations performed on personal data, such as collection, recording, organisation, structuring, storage, adaptation, retrieval, consultation, use, disclosure by transmission, dissemination, combination, erasure or destruction of personal data.*
 

- Yes *(please give examples)* (1) ________________________________________________
- No (2)

*Display This Question:*

*If Q95 = Yes <em>(please give examples)</em>*

Q96 When the ethics committee processes personal data in the scope of its activities, how should it be characterised under the GDPR?

- **As a data controller:** it determines the purposes and means of the processing of personal data, i.e. why and how personal data should be processed (1)
- **As a joint controller:** when two or more controllers jointly determine the purposes and means of processing of personal data (2)
- **As a data processor:** it processes personal data on behalf of a data controller (3)

| Page Break |  |
| --- | --- |

Q97 **Topic 2. Role of ethics committees in GDPR compliance**

Q98
**Does your ethics committee suggest what legal basis and/or special condition under GDPR should be used for the valid processing of personal data for primary use in research?**
  
*In this survey,****"primary use" of personal data*** *for research means the use of data directly collected and used for the purposes of clinical studies. For example, primary use purposes would include projects that were named at the time of data collection.*
 
***A valid legal ground is needed to process personal data.*** *Pursuant to the GDPR, there are six legal bases, specified in Article 6(1). Additionally,* ***if special categories of data*** *(such as data concerning health*- medical history, results of examinations and treatments etc*) are processed, there needs to be* ***a special condition under*** *Article 9(2) GDPR.*

- Yes (1)
- No (2)

*Display This Question:*

*If Q98 = Yes*

Q99 **Prior to the COVID-19 pandemic**, **how often** did your ethics committee advise that each **legal basis (Article 6(1))** should be used for research? *(By hovering over the legal bases, you can find a short explanation and a reference to the law)*

|  | Never (1) | Sometimes (2) | About half the time (3) | Most of the time (4) | Always (5) |
| --- | --- | --- | --- | --- | --- |
| Consent (1) |  |  |  |  |  |
| Contract (2) |  |  |  |  |  |
| Legal obligation (3) |  |  |  |  |  |
| Vital interests (4) |  |  |  |  |  |
| Public interest (5) |  |  |  |  |  |
| Legitimate interests (6) |  |  |  |  |  |

*Display This Question:*

*If Q98 = Yes*

Q100 Prior to the COVID-19 pandemic, how often did your ethics committee advise that each of the listed below **special conditions** (Article 9(2)) for the processing of special categories of personal data (such as data concerning health) should be used for research? *(By hovering over the conditions, you can find a short explanation and a reference to the law)*

|  | Never (1) | Sometimes (2) | About half the time (3) | Most of the time (4) | Always (5) |
| --- | --- | --- | --- | --- | --- |
| Explicit consent (1) |  |  |  |  |  |
| Vital interests (2) |  |  |  |  |  |
| Personal data manifestly made public (3) |  |  |  |  |  |
| Substantial public interest (4) |  |  |  |  |  |
| Preventive or occupational medicine (5) |  |  |  |  |  |
| Public interest in the area of public health (6) |  |  |  |  |  |
| Scientific research (7) |  |  |  |  |  |

*Display This Question:*

*If Q98 = Yes*

Q102 If you **indicated that your ethics committee advises "Most of the time" and "Always"**on the use of some legal bases and/or special conditions, please answer:  **During the COVID-19 pandemic**, did you suggest different legal bases/special conditions than the ones specified above?

- Yes *(please give examples)* (1) ________________________________________________
- No, there was no difference in the advice given prior to and during the COVID-19 pandemic (2)

*Display This Question:*

*If Q98 = Yes*

Q101 Does your ethics committee **suggest different** legal bases and/or special conditions to **commercial sponsors** of clinical studies **in comparison to non-commercial** sponsors?

- Yes *(please specify the legal bases and/or special conditions that you suggest to commercial and non-commercial sponsors respectively)* (1) ________________________________________________
- No (2)

Q103
Does your ethics committee suggest **what legal basis and/or other condition** should be used for the valid processing of personal data **for secondary use in research**?

 *In this survey,****"secondary use" of personal data*** *for research means the re-use (further processing) of personal data that was initially collected and used for another purpose. For example, when health data which was originally collected in the scope of care or in a previous clinical study is used for the conduct of another study, this would be considered secondary use.*

 *Personal data cannot be re-used if that would be incompatible with the initial purposes for collection and use.*

- Yes *(please specify)* (1) ________________________________________________
- No (2)

Q104 Does your ethics committees advise researchers what **specific techniques for anonymisation or pseudonymisation should be used when personal data** is processed for research?

- Yes *(please specify)* (1) ________________________________________________
- No (2)

Q105 **Prior to the COVID-19 pandemic**, what were the **biggest challenges for researchers** when providing **GDPR-related information** to study participants?

________________________________________________________________

Q106 **During the COVID-19 pandemic**, are the challenges concerning the provision of GDPR-related information the same or different?

- The same as prior to the COVID-19 pandemic (1)
- Different *(please specify)* (2) ________________________________________________

Q107 **Prior to the pandemic**, which **topics** do you think that researchers considered the most challenging? 
 
You can also specify additional challenges that were not listed (box "Other").

|  | Never (1) | Sometimes (2) | About half the time (3) | Most of the time (4) | Always (5) |
| --- | --- | --- | --- | --- | --- |
| Choice of legal basis for primary use of personal data (1) |  |  |  |  |  |
| Secondary use of personal data (2) |  |  |  |  |  |
| Providing information to study participants (3) |  |  |  |  |  |
| Communication with ethics committees (4) |  |  |  |  |  |
| Lack of legal harmonisation in case of multi-site studies in different countries (5) |  |  |  |  |  |
| Other *(please specify)* (6) |  |  |  |  |  |

Q108 **During the pandemic**, which **topics** do you think that researchers consider the most challenging?
 
You can also specify additional challenges that were not listed (box "Other").

|  | Never (1) | Sometimes (2) | About half the time (3) | Most of the time (4) | Always (5) |
| --- | --- | --- | --- | --- | --- |
| Choice of legal basis for primary use of personal data (1) |  |  |  |  |  |
| Secondary use of personal data (2) |  |  |  |  |  |
| Providing information to study participants (3) |  |  |  |  |  |
| Communication with ethics committees (4) |  |  |  |  |  |
| Lack of legal harmonisation in case of multi-site studies in different countries (5) |  |  |  |  |  |
| Other *(please specify)* (6) |  |  |  |  |  |

| 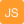 |
| --- |

Q109 In your view, do **commercial and non-commercial sponsors** of clinical studies experience different challenges?

- Yes *(please give examples)* (1) ________________________________________________
- No (2)
- I do not know (3)

| Page Break |  |
| --- | --- |

Q110 **Topic 3. Impact of the COVID-19 pandemic on the activities of ethics committees**

Q111 What was the impact of the COVID-19 pandemic on the work of your ethics committee?

|  | Not at all (1) | Somewhat (2) | Significantly (3) | Very significantly (4) |
| --- | --- | --- | --- | --- |
| The pandemic **positively impacted** our work *(please provide examples)* (1) |  |  |  |  |
| The pandemic **negatively impacted** our work *(please provide examples)* (2) |  |  |  |  |

Q112 **Prior to the pandemic, how often did your ethics committee receive questions about data protection issues?**

|  | Never (1) | Sometimes (2) | About half the time (3) | Most of the time (4) | Always (5) |
| --- | --- | --- | --- | --- | --- |
| The ethics committee received questions about data protection: (1) |  |  |  |  |  |

| 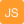 |
| --- |

Q113 **How did the COVID-19 pandemic affect the number of questions about data protection issues?**

|  | Decreased greatly (1) | Decreased slightly (2) | Stayed the same (3) | Increased slightly (4) | Increased greatly (5) |
| --- | --- | --- | --- | --- | --- |
| During the pandemic, the number of questions about data protection: (6) |  |  |  |  |  |

| Page Break |  |
| --- | --- |

Q114 Closing questions to Part I. Compliance with the General Data Protection Regulation for clinical studies

Q115 Do you want to mention an additional point of concern regarding data protection, not covered in this survey?

________________________________________________________________

| 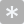 |
| --- |

Q116 Please share your email address, if you would like to participate in a follow-up interview that will explore in more depth the issues related to data protection.

________________________________________________________________

| 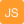 |
| --- |

Q117 You reached the end of Part I. The next set of questions deals with eConsent.

**End of Block: Part I. Ethics committees**

**Start of Block: Part II. Ethics committees**

Q118 **Part II. General aspects related to (electronic) informed consent**

Q119 **Topic 1. General question**

| 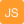 |
| --- |

Q120 What would be the ideal means of communication, regardless of the COVID-19 pandemic, to inform research participants about the objectives and conduct of a clinical study (e.g. study procedures, risks, benefits...)?

________________________________________________________________

________________________________________________________________

________________________________________________________________

________________________________________________________________

________________________________________________________________

| Page Break |  |
| --- | --- |

Q121 **Topic 2. Electronic informed consent**

Q122
Does national law or policy of the country in which your ethics committee operates, provide a definition of electronic informed consent?

- Yes *(please specify)* (1) ________________________________________________
- No (2)
- I do not know (3)

*Display This Question:*

*If Q122 = No*

*Or Q122 = I do not know*

Q123 What do you understand by electronic informed consent?

________________________________________________________________

________________________________________________________________

________________________________________________________________

________________________________________________________________

________________________________________________________________

Q124 Which functionalities should be part of an electronic informed consent platform (which is a platform enabling research participants to give and manage their electronic informed consent)? 
*(Multiple answers possible)*

- Providing research study information in an interactive and dynamic way to (potential) research subjects (1)
- Obtaining and documenting the signature of the research subjects (2)
- The return of research results to the research subjects (3)
- The possibility to reconsent research subjects (4)
- Other *(please specify)* (5) ________________________________________________

Q125
Is it legally allowed in the country where your ethics committee is based to **provide study-related information** to research participants via electronic means, before obtaining their informed consent?

- Yes (1)
- Only under certain conditions *(please specify)* (3) ________________________________________________
- No (4)
- I do not know (5)

Q126
Is it legally allowed in the country where your ethics committee is based to obtain research participants' informed **consent** via electronic means?

- Yes (1)
- Only under certain conditions *(please specify)* (4) ________________________________________________
- No (2)
- I do not know (3)

*Display This Question:*

*If Q126 = Yes*

*Or Q126 = Only under certain conditions <em>(please specify)</em>*

Q127 Which **signature(s)** is/are legally allowed to obtain the research participants' informed consent? 
*(You can find the definitions of the different signatures by hovering over the answer options - Multiple answers possible)*

- Simple or basic electronic signature (1)
- Advanced electronic signature (2)
- Qualified advanced electronic signature (3)
- I do not know (4)
- Other *(please specify)* (5) ________________________________________________

Q128 Which laws or regulations regulate (i.e. allow or prohibit) the use of electronic informed consent?

________________________________________________________________

________________________________________________________________

________________________________________________________________

________________________________________________________________

________________________________________________________________

| 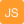 |
| --- |

Q129 Did your ethics committee review electronic informed consent in the past?

- Yes (1)
- No (2)
- I do not know (3)

*Display This Question:*

*If Q129 = Yes*

Q130 In your experience, was the review process of electronic informed consent more **complex**, compared to the review process of paper-based informed consent?

- Yes *(please specify)* (1) ________________________________________________
- No (2)

| Page Break |  |
| --- | --- |

Q131 **Topic 3. Informed consent during the COVID-19 pandemic**

Q132 Alternative methods were recommended, for example by the European Medicines Agency, to **re-consent for already included research participants**, e.g. due to protocol changes. These methods include:   Method 1: Obtaining oral consent (e.g. via phone or video-calls), supplemented with e-mail confirmation.   Method 2: Obtaining oral consent (e.g. via phone or video-calls). An appropriately signed and dated informed consent form should be obtained from the research participant as soon as possible.   Method 3: Using validated electronic systems e.g. electronic informed consent.   How useful are the alternative methods to re-consent for **already included research participants**?

|  | Not useful at all (1) | Slightly useful (2) | Moderately useful (3) | Extremely useful (6) |
| --- | --- | --- | --- | --- |
| **Method 1** (1) |  |  |  |  |
| **Method 2** (2) |  |  |  |  |
| **Method 3** (3) |  |  |  |  |

*Display This Question:*

*If Q132 = Not useful at all*

*Or Q132 = Slightly useful*

Q133 Please specify why the recommended method(s) is/are not useful at all or slightly useful

________________________________________________________________

________________________________________________________________

________________________________________________________________

________________________________________________________________

________________________________________________________________

*Display This Question:*

*If Q132 = Moderately useful*

*Or Q132 = Extremely useful*

Q134 Please specify why the recommended method(s) is/are moderately or extremely useful

________________________________________________________________

________________________________________________________________

________________________________________________________________

________________________________________________________________

________________________________________________________________

| 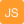 |
| --- |

Q135 Specific methods were recommended, for example by the European Medicines Agency, when obtaining informed consent in case **COVID-19 patients** are involved. 

 **Method 1**: If written consent by the research participant is not possible, consent could be given orally by the research participant in the presence of an impartial witness. In such cases, the witness is required to sign and date the informed consent form and the investigator is expected to record how the impartial witness was selected.

 

Method 2: The research participant and the person obtaining consent sign and date separate informed consent forms (i.e. one informed consent form signed and dated by the participant and another consent form signed and dated by the researcher). An appropriately signed and dated informed consent form should be obtained from the research participant later, as soon as possible.
 
Method 3: Using validated electronic systems, e.g. electronic informed consent.
 
How useful are the alternative methods for obtaining informed consent of **COVID-19 patients**?

|  | Not useful at all (1) | Slightly useful (2) | Moderately useful (3) | Extremely useful (4) |
| --- | --- | --- | --- | --- |
| **Method 1** (1) |  |  |  |  |
| **Method 2** (2) |  |  |  |  |
| **Method 3** (3) |  |  |  |  |

*Display This Question:*

*If Q135 = Not useful at all*

*Or Q135 = Slightly useful*

Q136 Please specify why the recommended method(s) is/are not useful at all or slightly useful

________________________________________________________________

________________________________________________________________

________________________________________________________________

________________________________________________________________

________________________________________________________________

*Display This Question:*

*If Q135 = Moderately useful*

*Or Q135 = Extremely useful*

Q137 Please specify why the recommended method(s) is/are moderately or extremely useful

________________________________________________________________

________________________________________________________________

________________________________________________________________

________________________________________________________________

________________________________________________________________

| Page Break |  |
| --- | --- |

**End of Block: Part II. Ethics committees**

**Start of Block: Introductory questions physicians**

Q138 Are you a member of the IMI-2 consortium Corona Accelerated R&D in Europe (CARE)? *

- Yes (1)
- No (2)

Q139 Where is your organisation based? *

▼ Austria (1) ... United Kingdom (28)

Q140 In which country are you involved in clinical studies? *

- In the country where I am based (national studies) (1)
- In several countries (international studies), *please specify in which countries* (2) ________________________________________________

Q141 Please select the answer that is applicable to you - I was/am involved in: *

- **COVID-19 studies:** clinical studies that investigate a COVID-19 medicine, diagnostic product or device, and/or vaccine (1)
- **Non-COVID-19 studies**: clinical studies that investigate a medicine, diagnostic product or device, and/or vaccine for other medical conditions (e.g. cancer) (2)

Q142 According to applicable legislation, there are different types of clinical studies. Below we list several types of clinical studies (you can find the definitions by hovering over their names).

 **Please select the type of studies in which you are involved most often. ***
 *(Multiple answers possible)*

- Interventional clinical trials (1)
- Non-interventional clinical trials (2)
- Clinical investigation of a medical device (3)
- Clinical performance study of an in vitro diagnostic medical device (5)

| 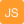 |
| --- |

Q143 Have you conducted a registry-based trial (i.e. an investigation of a research question which uses the infrastructure of a new or an existing registry for patient recruitment and/or data collection)? *

- Yes (1)
- No (2)
- I do not know (3)

**End of Block: Introductory questions physicians**

**Start of Block: Part I. Physicians**

Q144 **Part I. Compliance with the General Data Protection Regulation for clinical studies**

Q145 Topic 1. General questions

Q146 Do you have a formal training in data protection? *

- Yes (1)
- No (2)

Q147 How could knowledge about data protection among investigators/physicians be enhanced?

________________________________________________________________

| 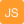 |
| --- |

Q148 Do you think that investigators have different responsibilities regarding the compliance with the GDPR depending on whether a clinical study is commercially sponsored, non-commercially sponsored, or initiated by an individual investigator?

|  | Investigators are **NOT responsible** for GDPR compliance (1) | Investigators **MAY BE responsible** for GDPR compliance in some cases (2) | Investigators are **FULLY responsible** for GDPR compliance (3) | *I do not know* (4) |
| --- | --- | --- | --- | --- |
| **Commercial study** (sponsored by a pharmaceutical company) (1) |  |  |  |  |
| **Non-commercial study** (for instance, sponsored by a research hospital) (2) |  |  |  |  |
| **Study initiated by an individual investigator** (3) |  |  |  |  |

| Page Break |  |
| --- | --- |

Q149 **Topic 2. Prospective (primary) use of personal data for research purposes**

 Below, you can find extra information that will make it easier to complete the survey.

 *In this survey,* **prospective (primary) use***of personal data for research means the use of data* ***directly collected*** *and used for the purposes of clinical studies. For example, primary use purposes would include projects that were named at the time of data collection.*
 
***"Personal data"****is any information about a particular natural person that allows, or could allow identifying the person. Examples of personal data include name and surname, home address.*
*There are also* ***special categories of personal data****, such as patients' health and genetic data (e.g. medical history, results of examinations and treatments, etc). Special categories of data are considered to be more sensitive, and the GDPR gives them extra protection.* 

 **"Processing" of personal data** covers a wide range of operations performed on personal data, such as collection, recording, organisation, structuring, storage, adaptation, retrieval, consultation, use, disclosure by transmission, dissemination, combination, erasure or destruction of personal data.

| 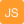 |
| --- |

Q150
**During the lifecycle of a clinical study**, there are **two main categories of processing operations**:
- Processing operations purely related to RESEARCH purposes
- Processing operations related to SAFETY purposes (for instance, notification of adverse events or serious adverse events to competent authorities)
 A valid legal basis is needed to process personal data. Pursuant to the GDPR, there are six legal bases, specified in **Article 6(1)**. Additionally, if **special categories of data (such as data concerning health)** are processed, there needs to be a special condition under **Article 9(2)** GDPR.
 (By hovering over the paragraph above, you can see a summary of these legal bases and special conditions.)

 **How many legal bases and special conditions do you rely on in the scope of one clinical study?**

- One legal basis + one special condition (for both processing operations related to research and related to safety purposes) (1)
- Two legal bases + two special conditions (one for processing operations related to research, and one for operations related to safety purposes) (2)
- I do not know (5)

*Display This Question:*

*If Q150 = Two legal bases + two special conditions (one for processing operations related to research, and one for operations related to safety purposes)*

*And Q146 = Yes*

Q151 For prospective (primary) use of personal data for RESEARCH purposes, please indicate how frequently you rely on each of the six legal bases available under Article 6(1) GDPR. *(By hovering over the legal bases, you can find a short explanation and a reference to the law)*

|  | Never (1) | Sometimes (2) | About half the time (3) | Most of the time (4) | Always (5) | *I do not know* (6) |
| --- | --- | --- | --- | --- | --- | --- |
| Consent (1) |  |  |  |  |  |  |
| Contract (2) |  |  |  |  |  |  |
| Legal obligation (3) |  |  |  |  |  |  |
| Vital interests (4) |  |  |  |  |  |  |
| Public interest (5) |  |  |  |  |  |  |
| Legitimate interests (6) |  |  |  |  |  |  |

*Display This Question:*

*If Q150 = Two legal bases + two special conditions (one for processing operations related to research, and one for operations related to safety purposes)*

*And Q146 = Yes*

Q152
For prospective (primary use) of special categories of personal data (such as data concerning health - medical history, results of examinations and treatments etc) for RESEARCH purposes, please indicate how frequently you rely on each of the listed below special conditions under Article 9(2) GDPR. *(By hovering over the conditions, you can find a short explanation and a reference to the law)*

|  | Never (1) | Sometimes (2) | About half the time (3) | Most of the time (4) | Always (5) | *I do not know* (6) |
| --- | --- | --- | --- | --- | --- | --- |
| Explicit consent (1) |  |  |  |  |  |  |
| Vital interests (2) |  |  |  |  |  |  |
| Personal data manifestly made public (3) |  |  |  |  |  |  |
| Substantial public interest (4) |  |  |  |  |  |  |
| Preventive or occupational medicine (5) |  |  |  |  |  |  |
| Public interest in the area of public health (6) |  |  |  |  |  |  |
| Scientific research (7) |  |  |  |  |  |  |

*Display This Question:*

*If Q150 = Two legal bases + two special conditions (one for processing operations related to research, and one for operations related to safety purposes)*

*And Q146 = Yes*

Q153 If you indicated that you rely on some legal bases/special conditions "Most of the time" and "Always" for the primary use of personal data for RESEARCH purposes, please answer:
 
During the COVID-19 pandemic, did you use different legal bases/special conditions?

- Yes, during the pandemic, I relied on different legal bases/special conditions. *(Please specify why and provide examples)* (1) ________________________________________________
- No (2)
- I do not know (3)

*Display This Question:*

*If Q150 = One legal basis + one special condition (for both processing operations related to research and related to safety purposes)*

*And Q146 = Yes*

Q154 For prospective (primary) use of personal data for RESEARCH and SAFETY purposes, please indicate how frequently you rely on each of the six legal bases available under Article 6(1) GDPR. *(By hovering over the legal bases, you can find a short explanation and a reference to the law)*

|  | Never (1) | Sometimes (2) | About half the time (3) | Most of the time (4) | Always (5) | *I do not know* (6) |
| --- | --- | --- | --- | --- | --- | --- |
| Consent (1) |  |  |  |  |  |  |
| Contract (2) |  |  |  |  |  |  |
| Legal obligation (3) |  |  |  |  |  |  |
| Vital interests (4) |  |  |  |  |  |  |
| Public interest (5) |  |  |  |  |  |  |
| Legitimate interests (6) |  |  |  |  |  |  |

*Display This Question:*

*If Q150 = One legal basis + one special condition (for both processing operations related to research and related to safety purposes)*

*And Q146 = Yes*

Q155 For the prospective (primary) use of special categories of personal data (such as data concerning health - medical history, results of examinations and treatments etc) for RESEARCH and SAFETY purposes, please indicate how frequently you rely on each of the listed below special conditions under Article 9(2) GDPR. *(By hovering over the conditions, you can find a short explanation and a reference to the law)*

|  | Never (1) | Sometimes (2) | About half the time (3) | Most of the time (4) | Always (5) | *I do not know* (6) |
| --- | --- | --- | --- | --- | --- | --- |
| Explicit consent (1) |  |  |  |  |  |  |
| Vital interests (2) |  |  |  |  |  |  |
| Personal data manifestly made public (3) |  |  |  |  |  |  |
| Substantial public interest (4) |  |  |  |  |  |  |
| Preventive or occupational medicine (5) |  |  |  |  |  |  |
| Public interest in the area of public health (6) |  |  |  |  |  |  |
| Scientific research (7) |  |  |  |  |  |  |

*Display This Question:*

*If Q150 = One legal basis + one special condition (for both processing operations related to research and related to safety purposes)*

*And Q146 = Yes*

Q156
If you indicated that you rely on some legal bases/special conditions "Most of the time" and "Always" for the use of personal data for RESEARCH and SAFETY purposes, please answer: 
 
During the COVID-19 pandemic, did this change?

- Yes, during the pandemic, I relied on different legal bases/special conditions. *(Please specify why and provide examples)* (1) ________________________________________________
- No (2)
- I do not know (3)

*Display This Question:*

*If Q150 = One legal basis + one special condition (for both processing operations related to research and related to safety purposes)*

*And Q146 = Yes*

*And Q148 = <strong>Study initiated by an individual investigator</strong> [ Investigators are FULLY responsible for GDPR compliance ]*

Q157 **If you indicated "Most of the time" or "Always"** to any of the legal bases/special conditions listed below, please provide **a reference to the national law**, which implements the relevant provision. 

- **Legal obligation:** processing is necessary for compliance with a legal obligation (Article 6(1)(c)) (1) ________________________________________________
- **Public interest:** processing is necessary for the performance of a task carried out in the public interest (Article 6(1)(e)) (2) ________________________________________________
- **Substantial public interest:** processing is necessary for reasons of substantial public interest (Article 9(2)(g) (3) ________________________________________________
- **Public interest in the area of public health:** processing is necessary for public interest in the area of public health (Article 9(2)(i)) (4) ________________________________________________
- **Scientific research:** processing is necessary for archiving purposes in the public interest, scientific or historical research purposes or statistical purposes (Article 9(2)(j)) (5) ________________________________________________
- I do not know (6)
- Not applicable to me (7)

| 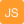 |
| --- |

Q158
*Data controller, pursuant to the GDPR, is the natural or legal person which determines the purposes and means of the data processing, i.e. the controller decides 'why' and 'how' the personal data should be processed.*
 
**In an international clinical study, who** would be the **data controller for the processing operations related to SAFETY purposes,** which are **conducted at an investigator site** (healthcare provider) **other than the one in which you work at?**

- The sponsor of the clinical study (1)
- The investigator site from which the safety data originates (2)
- The investigator site where I work (3)
- Both the sponsor of the clinical study and the investigator site from which the safety data originates (4)
- Both investigator site from which the safety data originates and the investigator site where I work (5)
- I do not know (6)

| Page Break |  |
| --- | --- |

Q159 **Topic 3: Retrospective (secondary) use of personal data**

 *In this survey***, retrospective (secondary) use***of personal data for research means the re-use (further processing) of personal data that was initially collected and used for another purpose.*
 
*For example, when health data which was originally collected in the scope of care or in a previous clinical study, is used for the conduct of another study, this would be considered secondary use. 

 Personal data cannot be re-used if that would be incompatible with the initial purposes for collection and use.*

Q160 When you re-use personal data from a previous project, how often do you rely on the presumption of compatibility for research (Article 5(1)(b) GDPR)?

 The presumption of compatibility means that the re-use of personal data for scientific research purposes is considered compatible with the initial purposes, provided that appropriate safeguards are in place.

|  | Never (1) | Sometimes (2) | About half the time (3) | Most of the time (4) | Always (5) | *I do not know* (6) |
| --- | --- | --- | --- | --- | --- | --- |
| For the re-use of personal data, I rely on the presumption of compatibility: (1) |  |  |  |  |  |  |

*Display This Question:*

*If Q160 = Always*

*Or Q160 = Most of the time*

Q161 Which statement do you agree with?

- When I re-use personal data for research purposes, **I can rely on the same legal basis** as for the initial (primary) use (1)
- **I need to have a new legal basis** in order to re-use personal data for research purposes (2)
- **Both options** listed above are valid (3)
- **I do not think that a legal basis is required at all** for the re-use of personal data for research (4)
- No opinion (5)

Q162 **Compatibility assessment**
 Personal data can be re-used for another purpose (e.g. a new study) after checking that the new purpose is compatible with the original purpose for which the data was collected. This check is known as the compatibility assessment (Article 6(4)), for which the GDPR established five elements that should be considered. *(You can see a summary of these elements by hovering over the text above)*
 
 **How often does your organisation conduct the compatibility assessment when re-using personal data for research?**

|  | Never (1) | Sometimes (2) | About half the time (3) | Most of the time (4) | Always (5) | *I do not know* (6) |
| --- | --- | --- | --- | --- | --- | --- |
| I conduct the compatibility assessment: (6) |  |  |  |  |  |  |

*Display This Question:*

*If Q162 = Always*

*Or Q162 = Most of the time*

*Or Q162 = I conduct the compatibility assessment: [ Always ]*

*Or Q162 = I conduct the compatibility assessment: [ Most of the time ]*

Q163 Please rank **from most (1) to least important (5)**the elements of the compatibility assessment. You can do so by dragging and dropping each item.

______ **Any link between the purposes** for which the personal data have been collected and the purposes of the intended further processing. (1)

______ **The context** in which the personal data have been collected (what is the relationship between your organisation and the individual). (2)

______ **The nature of the personal data**, in particular whether special categories of personal data are processed (such as data concerning health). (3)

______ **The possible consequences** of the intended further processing for data subjects (how it will impact individuals). (4)

______ The existence of **appropriate safeguards** (for instance encryption or pseudonymisation). (5)

| 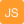 |
| --- |

Q164 During the COVID-19 pandemic, did anything change in the way that you process personal data for secondary use?

- Yes *(please specify how)* (1) ________________________________________________
- No (2)

| Page Break |  |
| --- | --- |

Q165 **Topic 4. Transparency**

Q166 **When you collect personal data** for **prospective** (primary) use research purposes, do you clearly inform study participants about the categories of personal data (such as age, sex, drug test results, etc), that are being collected?

- Yes (1)
- No (2)
- I do not know (3)

Q167 **Prior to the COVID-19 pandemic**, what were the biggest challenges that you experienced when providing GDPR-related information to study participants?

________________________________________________________________

| 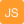 |
| --- |

Q168 **During the COVID-19 pandemic**, are the biggest challenges still the same as the ones you listed above, or different?

- The same (1)
- Different *(please specify)* (2) ________________________________________________

| Page Break |  |
| --- | --- |

Q169 **Topic 5. Communication with ethics committees**

Q170 Do you consider that the GDPR establishes a special role for ethics committees when it comes to the way that research organisations should process personal data for scientific research?

- Yes *(please specify)* (1) ________________________________________________
- No (2)

*Display This Question:*

*If Q170 = No*

Q171 Do you think that ethics committees should have such a special role?

- Yes *(please specify why)* (1) ________________________________________________
- No *(please specify why)* (2) ________________________________________________

Q172 Do you receive comments from ethics committees in relation to GDPR-related text in your study protocols? For instance, asking for the deletion or inclusion of specific text.

- Yes *(please provide examples)* (1) ________________________________________________
- No (2)
- I do not know (3)

*Display This Question:*

*If Q172 = Yes<em> (please provide examples)</em>*

Q173 Do you think that if you follow some of these ethics committees' comments, your study may become non-compliant with the GDPR?

- Yes *(please specify)* (1) ________________________________________________
- No (2)
- I do not know (3)

Q174 Do ethics committees sometimes insist on the use of a specific legal basis under GDPR for the prospective (primary) use of personal data for research?

- Yes *(please provide examples)* (1) ________________________________________________
- No (2)
- I do not know (3)

Q175 Do ethics committees sometimes insist on the use of a specific legal basis under GDPR for the retrospective (secondary use) of personal data for research?

- Yes *(please provide examples)* (1) ________________________________________________
- No (2)
- I do not know (3)

| 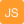 |
| --- |

Q176 Do ethics committees sometimes insist on the use of a specific technique for anonymisation or pseudonymisation of personal data?

- Yes *(please specify)* (1) ________________________________________________
- No (2)
- I do not know (3)

| Page Break |  |
| --- | --- |

Q177 **Topic 6. Challenges prior to and during the COVID-19 pandemic**

Q178 **Prior to the pandemic**, in relation to which of the **topics** below did you experience **challenges**? 
 
You can also specify additional challenges that were not listed (box "Other").

|  | Never (1) | Sometimes (2) | About half the time (3) | Most of the time (4) | Always (5) |
| --- | --- | --- | --- | --- | --- |
| Choice of legal basis for prospective (primary) use of personal data (1) |  |  |  |  |  |
| Retrospective (secondary) use of personal data (2) |  |  |  |  |  |
| Providing information to study participants (3) |  |  |  |  |  |
| Communication with ethics committees (4) |  |  |  |  |  |
| Lack of legal harmonisation in case of multi-site studies in different countries (5) |  |  |  |  |  |
| Other *(please specify)* (6) |  |  |  |  |  |

| 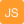 |
| --- |

Q179 **During the pandemic**, in relation to which of the **topics** below did you experience **challenges**?
 
You can also specify additional challenges that were not listed (box "Other").

|  | Never (1) | Sometimes (2) | About half the time (3) | Most of the time (4) | Always (5) |
| --- | --- | --- | --- | --- | --- |
| Choice of legal basis for prospective (primary) use of personal data (1) |  |  |  |  |  |
| Retrospective (secondary) use of personal data (2) |  |  |  |  |  |
| Providing information to study participants (3) |  |  |  |  |  |
| Communication with ethics committees (4) |  |  |  |  |  |
| Lack of legal harmonisation in case of multi-site studies in different countries (5) |  |  |  |  |  |
| Other *(please specify)* (6) |  |  |  |  |  |

| Page Break |  |
| --- | --- |

Q180 Closing questions to Part I. Compliance with the General Data Protection Regulation for clinical studies

Q181 Do you want to mention an additional point of concern regarding data protection, not covered in this survey?

________________________________________________________________

| 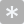 |
| --- |

Q182 Please share your email address, if you would like to participate in a follow-up interview that will explore in more depth the issues related to data protection.

________________________________________________________________

| 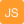 |
| --- |

Q183 You reached the end of Part I. The next set of questions deals with eConsent.

**End of Block: Part I. Physicians**

**Start of Block: Part II. Physicians**

Q184 **Part II. General aspects related to (electronic) informed consent**

Q185 **Topic 1. General question**

| 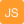 |
| --- |

Q186 What would be the ideal means of communication, regardless of the COVID-19 pandemic, to inform research participants about the objectives and conduct of a clinical study (e.g. study procedures, risks, benefits...)?

________________________________________________________________

________________________________________________________________

________________________________________________________________

________________________________________________________________

________________________________________________________________

| Page Break |  |
| --- | --- |

Q187 **Topic 2. Electronic informed consent**

Q188 Do you have experience with **informing research participants and obtaining their informed consent** via electronic means?

- Yes (1)
- No (2)

*Display This Question:*

*If Q188 = Yes*

Q189 For which clinical study(ies) do you use electronic means to inform research participants and obtain their consent? 
*(You can find more information about each study type by hovering over the answer options - Multiple answers possible)*

- Interventional clinical trials (1)
- Non-interventional clinical trials (2)
- Clinical investigation of a medical device (3)
- Clinical performance study of an in vitro diagnostic medical device (4)

*Display This Question:*

*If Q188 = Yes*

Q190 Which electronic mean(s) do you use to inform research participants and obtain their consent? 
*(Multiple answers possible)*

- Phone (1)
- Tablet (2)
- Computer (3)
- Other *(please specify)* (4) ________________________________________________

*Display This Question:*

*If Q140 = In the country where I am based (national studies)*

Q191 Does national law or policy of the country in which you operate, provide a definition of electronic informed consent?

- Yes *(please specify)* (1) ________________________________________________
- No (2)
- I do not know (3)

*Display This Question:*

*If Q140 = In several countries (international studies), <em>please specify in which countries</em>*

Q192
Does national law or policy of at least one of the countries where you operate, provide a definition of electronic informed consent?

- Yes *(please specify which country(ies) and definition(s))* (1) ________________________________________________
- No (2)
- I do not know (3)

*Display This Question:*

*If Q191 = No*

*Or Q191 = I do not know*

*Or Q192 = I do not know*

*Or Q192 = No*

Q193 What do you understand by electronic informed consent?

________________________________________________________________

________________________________________________________________

________________________________________________________________

________________________________________________________________

________________________________________________________________

Q194 Which **functionalities** should be part of an electronic informed consent platform (which is a platform enabling research participants to give and manage their electronic informed consent)? 
*(Multiple answers possible)*

- Providing research study information in an interactive and dynamic way to (potential) research subjects (1)
- Obtaining and documenting the signature of the research subjects (2)
- The return of research results to the research subjects (3)
- The possibility to reconsent research subjects (4)
- Other *(please specify)* (5) ________________________________________________

*Display This Question:*

*If Q140 = In the country where I am based (national studies)*

Q195
Is it legally allowed in the country where you operate to **provide study-related information** to research participants via electronic means, before obtaining their informed consent?

- Yes (1)
- Only under certain conditions *(please specify)* (3) ________________________________________________
- No (4)
- I do not know (5)

*Display This Question:*

*If Q140 = In several countries (international studies), <em>please specify in which countries</em>*

Q196
Is it legally allowed in at least one of the countries where you operate to provide study-related information to research participants via electronic means, before obtaining their informed consent?

- Yes *(please specify which country(ies))* (1) ________________________________________________
- Only under certain conditions *(please specify which country(ies) and conditions)* (2) ________________________________________________
- No (3)
- I do not know (4)

*Display This Question:*

*If Q140 = In the country where I am based (national studies)*

Q197
Is it legally allowed in the country where you operate to obtain research participants' informed **consent** via electronic means?

- Yes (1)
- Only under certain conditions *(please specify)* (2) ________________________________________________
- No (3)
- I do not know (4)

*Display This Question:*

*If Q140 = In several countries (international studies), <em>please specify in which countries</em>*

Q198 Is it legally allowed in at least one of the countries where you operate to obtain research participants' informed consent via electronic means?

- Yes *(please specify which country(ies))* (1) ________________________________________________
- Only under certain conditions *(please specify which country(ies) and conditions)* (2) ________________________________________________
- No (5)
- I do not know (6)

*Display This Question:*

*If Q197 = Yes*

*Or Q197 = Only under certain conditions<em> (please specify)</em>*

*Or Q198 = Yes<em> (please specify which country(ies))</em>*

*Or Q198 = Only under certain conditions<em> (please specify which country(ies) and conditions)</em>*

Q199 Which **signature(s)** is/are legally allowed to obtain the research participants' informed consent? 
*(You can find the definitions of the different signatures by hovering over the answer options - Multiple answers possible)*

- Simple or basic electronic signature (1)
- Advanced electronic signature (2)
- Qualified advanced electronic signature (3)
- I do not know (4)
- Other *(please specify)* (5) ________________________________________________

| 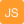 |
| --- |

Q200 Which laws or regulations regulate (i.e. allow or prohibit) the use of electronic informed consent?

________________________________________________________________

________________________________________________________________

________________________________________________________________

________________________________________________________________

________________________________________________________________

| Page Break |  |
| --- | --- |

Q201 **Topic 3. Informed consent during the COVID-19 pandemic**

Q202 Alternative methods were recommended, for example by the European Medicines Agency, to **re-consent for already included research participants**, e.g. due to protocol changes. These methods include:
 
**Method 1**: Obtaining oral consent (e.g. via phone or video-calls), supplemented with e-mail confirmation.
 
**Method 2**: Obtaining oral consent (e.g. via phone or video-calls). An appropriately signed and dated informed consent form should be obtained from the research participant as soon as possible.
 
**Method 3**: Using validated electronic systems, e.g. electronic informed consent.
 
Which method(s) did you employ during the COVID-19 pandemic? 
*(Multiple answers possible)*

- Method 1 (1)
- Method 2 (2)
- Method 3 (3)
- Other method *(please specify)* (4) ________________________________________________
- Not applicable (5)

Q203 How useful are the alternative methods to re-consent for **already included research participants**? *(You can find more information about each method by hovering over their names)*

|  | Not usefull at al (35) | Slightly useful (36) | Moderately useful (38) | Extremely useful (40) |
| --- | --- | --- | --- | --- |
| **Method 1** (24) |  |  |  |  |
| **Method 2** (25) |  |  |  |  |
| **Method 3** (26) |  |  |  |  |
| **Other *(if any)*** (27) |  |  |  |  |

*Display This Question:*

*If Q203 = Not usefull at al*

*Or Q203 = Slightly useful*

Q204 Please specify why the recommended method(s) is/are not useful at all or slightly useful

________________________________________________________________

________________________________________________________________

________________________________________________________________

________________________________________________________________

________________________________________________________________

*Display This Question:*

*If Q203 = Moderately useful*

*Or Q203 = Extremely useful*

Q205 Please specify why the recommended method(s) is/are moderately or extremely useful

________________________________________________________________

________________________________________________________________

________________________________________________________________

________________________________________________________________

________________________________________________________________

Q206
Specific methods were recommended, for example by the European Medicines Agency, when obtaining informed consent of **COVID-19 patients**. These methods include:
 
Method 1: If written consent by the research participant is not possible, consent could be given orally by the research participant in the presence of an impartial witness. In such cases, the witness is required to sign and date the informed consent form and the investigator is expected to record how the impartial witness was selected.
 
Method 2: The research participant and the person obtaining consent sign and date separate informed consent forms (i.e. one informed consent form signed and dated by the participant and another consent form signed and dated by the researcher). An appropriately signed and dated informed consent should be obtained from the research participant later, as soon as possible.
 
Method 3: Using validated electronic systems, e.g. electronic informed consent.
 
Which method(s) did you employ during the COVID-19 pandemic? 
*(Multiple answers possible)*

- Method 1 (1)
- Method 2 (2)
- Method 3 (3)
- Other method *(please specify)* (4) ________________________________________________
- Not applicable (5)

Q207 How useful are the alternative methods for obtaining informed consent of **COVID-19 patients**?
*(You can find more information about each method by hovering over their names)*

|  | Not useful at all (1) | Slightly useful (2) | Moderately useful (3) | Extremely useful (4) |
| --- | --- | --- | --- | --- |
| **Method 1 (8)** |  |  |  |  |
| **Method 2** (9) |  |  |  |  |
| **Method 3** (10) |  |  |  |  |
| **Other *(if any)*** (11) |  |  |  |  |

*Display This Question:*

*If Q207 = Not useful at all*

*Or Q207 = Slightly useful*

Q208 Please specify why the recommended method(s) is/are not useful at all or slightly useful

________________________________________________________________

________________________________________________________________

________________________________________________________________

________________________________________________________________

________________________________________________________________

*Display This Question:*

*If Q207 = Moderately useful*

*Or Q207 = Extremely useful*

Q209 Please specify why the recommended method(s) is/are moderately or extremely useful

________________________________________________________________

________________________________________________________________

________________________________________________________________

________________________________________________________________

________________________________________________________________

Q210 **Prior to the pandemic**, what were the biggest challenges that you experienced when providing research information to participants and obtaining consent?

________________________________________________________________

| 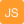 |
| --- |

Q211 **During the pandemic**, are these challenges the same, or different?

- The same (2)
- Different *(please specify why)* (3) ________________________________________________

| Page Break |  |
| --- | --- |
