# Supplementary material for: Challenges related to data protection in clinical research before and during the COVID-19 pandemic: An exploratory study
Source: Front Med (Lausanne). 2022 Oct 10;9:995689. doi: 10.3389/fmed.2022.995689 (PMC9589288; doi:10.3389/fmed.2022.995689)
Supplement: Supplementary file 2 [file Data_Sheet_2.DOCX]

Supplementary Material 2

# Interview guide

- **Present yourself (name and affiliation)**
- **Thank the interviewee** for their participation in the project.
- Explain **very** **briefly the purpose** of the interview, state its duration (apprx 1 h), and where the results will be implemented.
  - *This interview is the follow-up to an online survey. Both the interview and the survey are part of a study with which we want to gather evidence about the experience of relevant stakeholders involved in clinical research on key challenges and related possible solutions concerning the compliance with data protection rules prior to and during the COVID-19 pandemic.*
  - *The results will* ***be incorporated*** *in scientific publications, PhD dissertation, and will also serve to guide the IMI CARE consortium in being GDPR compliant.*
- Put the interviewee **at ease**:
  - *No wrong answers*
  - *Digitally recorded*
  - *Confidential, anonymous*
  - *Voluntary, do not have to answer anything they do not feel comfortable answering*
  - *They can stop the interview at any time, without having to give a reason*
- Ask the interviewee if they have any **questions** before the start of the interview.
- **Explain how the interview will proceed**, e.g*.:*

*We will start with some warming-up questions. Subsequently, we will focus on the research questions, using as a basis the online survey in which you have participated. We will look into primary and secondary use of personal data for clinical studies, transparency, data control, communication with ethics committees, and challenges encountered prior to and during the pandemic.*

- **Ask for permission to turn on the recording function.**

**Warming-up questions**:

- Can you tell me a little bit about yourself and your background?
- To what extent do you have experience with clinical research?

I. DPOs/legal experts

1. TOPIC 1: PRIMARY USE

**1.1 For participants who were directed to the pathway of questions that follows if answer ‘Two legal bases’ is chosen to Q13 and ‘Yes, it should be a combination’ to Q14:**

**1.** [Follow-up to survey Q15**] Please elaborate why, in your view, the organisation where you work at relies mostly on the following legal bases for the primary use of personal data for research purposes** [cite the legal basis which they indicated as “Always” and “Most of the time” used; ask further about the use of ‘consent’ (e.g. if they chose ‘never’ - why never]**?**

**2.** [Follow-up to Q16**] Please elaborate why, in your view, the organisation where you work at relies mostly on the following special conditions for the primary use of personal data for research purposes** [cite conditions which they indicated as “Frequently” and “Always” used; ask further about ‘consent’]**?**

**3.** [Follow-up to Q17**] Please elaborate why, in your view, during the pandemic your organisation changed the legal basis/condition they would normally rely on** [when answer ‘No’ was chosen in the survey]**?**

**1.2 For participants who were directed to the pathway of questions that follows if answer “Two legal bases” was chosen to Q13, and “No, it should not be a combination (I only apply Article 9(2) was chosen to Q14.**

**1.** [Follow-up to Q18**] Please elaborate why, in your view, the organisation where you work at relies mostly on the following special conditions for the primary use of personal data for research purposes** [cite the legal basis which they indicated as “Always” and “Most of the time” used; ask further about the use of ‘consent’ (e.g. if they chose ‘never’ - why never]**?**

**2.** [Follow-up to Q19**] Please elaborate why, in your view, during the pandemic**

- **your organisation changed the conditions they would normally rely on** [when answer "No” was chosen in the survey]**?**

**1.3 For participants who were directed to the pathway of questions that follows if answer “One legal basis” was chosen to Q13, and “Yes, it should not be a combination” was chosen to Q14.**

**1.** [Follow-up to Q20**] Please elaborate why, in your view, the organisation where you work at relies mostly on the following legal bases for the primary use of personal data for research and safety purposes** [cite the legal basis which they indicated as “Frequently” and “Always” used; ask further about the use of ‘consent’ (e.g. if they chose ‘never’ - why never]**?**

**2.** [Follow-up to Q21**] Please elaborate why, in your view, the organisation where you work at relies mostly on the following special conditions for the primary use of personal data for research and safety purposes** [cite conditions which they indicated as “Frequently” and “Always” used]**?**

**3.** [Follow-up to Q22**] Please elaborate why, in your view, during the pandemic**

- **your organisation changed the legal basis/condition they would normally rely on** [when answer "No” was chosen in the survey]**?**

**1.3. For participants who were directed to the pathway of questions that follows if answer “One legal basis” was chosen to Q13, and “No, it should not be a combination” was chosen to Q14**

**1.** [Follow-up to Q23**] Please elaborate why, in your view, the organisation where you work at relies mostly on the following special conditions for the primary use of personal data for research and safety purposes** [cite the legal basis which they indicated as “Always” and “Most of the time” used; ask further about the use of ‘consent’ (e.g. if they chose ‘never’ - why never]**?**

**2.** [Follow-up to Q24**] Please elaborate why, in your view, during the pandemic**

- **your organisation changed the conditions they would normally rely on** [when answer “No” was chosen in the survey]**?**

1. TOPIC 2: SECONDARY USE

**2.1** [Follow-up to Q29, if answers “Sometime”, “About half the time”, “Most of the time”, “Always” are chosen] **For the secondary use of personal data for research, how does your organisation apply the presumption of compatibility**?

**2.2** [Follow-up to Q29, if answer “Never” is chosen] **For the secondary use of personal data for research, please elaborate why your organisation does not rely on the presumption of compatibility**?

**3.1** [Follow-up to Q33, if answers “Sometime”, “About half the time”, “Most of the time”, “Always” are chosen ] **In your view, why does your organisation choose to conduct the compatibility assessment?**

**3.2** [Follow-up to Q33, if answer “Never” ] **In your view,** **why does your organisation not conduct the compatibility assessment?**

4. [Follow-up to Q34] **Please elaborate on the reasons why, according to you, the following elements** [to cite the participant’s answer in the survey] **of the compatibility assessment are the most important?**

5. **Do you think that the rules for the secondary use of personal data for scientific research are or should be applied differently when**:

- the original controller – meaning the controller who processed the data for primary use – re-uses it for further purposes
- another controller – meaning a controller to whom the personal data has been shared by the original controller – re-uses the data for further purposes?

Please elaborate (regardless of the answer: Yes/No)

**6. [Precision medicine question]** Precision medicine is a new therapeutic approach that promises to cure the incurable by choosing treatment based on the individual’s genetic profile. In practice, the precision medicine mechanism consists of a diagnostic device which is combined with a drug. The patient is tested so that his genetic makeup could be determined and that it could be measured whether he is likely to respond to the drug. There are new therapies, mainly in oncology at the moment, that only work on specific mutations.

**In your experience, are there any differences in complying with the principle of lawfulness – i.e. the choice of a legal basis for primary or secondary use – when the clinical study that is conducted by your organisation employs a precision medicine approach?**

- **If yes: please elaborate**
- **If no: please elaborate**

1. TOPIC 3: Transparency

1. When your organisation prospectively collects personal data for research purposes, how do they inform study participants about it?

2. How would you ideally provide information?

3. During the pandemic and lockdowns, was there a change in the way that your organisation provided information to study participants about the processing of their personal data?

4. How would you ideally provide information if there is another lockdown/a new pandemic?

5. In situation where your organisation re-uses personal data obtained from another data controller, do they inform the study participants about the re-use?

- If no: Why? *For instance, does your organisation rely on the exceptions in Article 14(5)(b) or Article 11 of the GDPR [the two provisions will be explained]?*
- If yes: How? *For instance, public announcement? Or do they inform the study participants with the assistance of the original data controller, based on contractual agreements?*

- if their organisation has never considered the assistance of the original data controller, explore why; would they; what do they think about the possibility?

6. [Precision medicine question] In your experience, are there any differences in complying with the principle of transparency when the clinical study that is conducted by your organisation employs a precision medicine approach?

- If yes: please elaborate
- If no: please elaborate

1. TOPIC 4: Data control

*It is through the data protection legal framework that individuals are empowered and receive control over their personal data (Recital 7 GDPR).*

1. What do you think about data control of individuals over their personal data in clinical research?

2. How do you think data control by individuals/participants could work in practice?

3. There is a new legislation on data sharing at the European level (called the Data Governance Act), wherein the concept ‘data altruism’ is proposed. This refers amongst others to ‘the consent by data subjects to process their personal data, for purposes of general interest, such as scientific research purposes or improving public services’. So persons giving consent to process their data for purposes of public interest. What is your view of newly proposed data altruism mechanism?

1. TOPIC 5: Communication with ethics committees

1. [Follow-up to Q45 and Q46]

- [if answer “Yes” to either Q] *For instance, does your organisation follow their advice? What happens if the organisation does not follow the advice?*

2. [Follow-up to Q47]

- [if answer “Yes”] **Please share your thoughts about situations where ethics committees would insist on the use of specific techniques for pseudo- or anonymisation?**
- Possible follow-up questions: *For instance, does your organisation follow their advice? What happens if the organisation does not follow the advice?*

3. **[Precision medicine question]** In your experience, are there any differences in the advice provided by the ethics committee on data protection issues, when the clinical study that is conducted by your organisation employs a precision medicine approach?

1. TOPIC 6: Challenges prior to and during the pandemic

1. [Follow-up to Q49] **In the survey, you indicated the following topics** [to insert based on the participant’s answer] **as the ones with which you and/or your organisation** **has experienced the most challenges prior to the pandemic. Could you please share concrete examples related to the topics?**

2. [Follow-up to Q49] Could you **please elaborate why these topics are the most challenging**, according to you?

3. [Follow-up to Q50] **In the survey, you indicated that the following topics** [to insert based on the participant’s answer] **as the ones with which you and/or organisation experienced the most challenges during the pandemic. Could you please share concrete examples related to the topics?**

4. [Follow-up to both Q49 and Q50]

- in case the challenges are the same: **Why, according to you?**
- in case the challenges are different: **Why, according to you?**

5. [Follow-up to Q50] **Could you please elaborate why these topics are the most challenging, according to you?**

6. [Follow-up to Q50] **What, do you think, is the cause of these challenges?**

7. [Follow-up to Q50] **How would you normally address them?**

8. [Follow-up to Q49 and 50] **How would you ideally address challenges experienced prior to and during the pandemic or ask to be addressed at EU/national level?**

9. [Precision medicine question] In your experience, are the challenges different, when the clinical study that is conducted by your organisation employs a precision medicine approach?

1. Closing questions

1. As a final question, do you have any further comments or issues which you think may be relevant for the compliance with the GDPR for clinical studies? If yes, which one(s)?

2. Do you have any questions for me?

2. Investigators

TOPIC 1: PRIMARY USE

**1.1 For participants who were directed to the pathway of questions that follows if answer ‘Two legal bases’ is chosen to Q150 and ‘Yes’ to Q146 “Do you have a formal training in data protection?”:**

**1.** [Follow-up to Q151**] Please elaborate why, in your view, you rely mostly on the following legal bases for the primary use of personal data for research purposes** [cite the legal basis which they indicated as “Always” and “Most of the time” used; ask further about the use of ‘consent’ (e.g. if they chose ‘never’ - why never]**?**

**2.** [Follow-up to Q152**] Please elaborate why, in your view, you rely mostly on the following special conditions for the primary use of personal data for research purposes** [cite conditions which they indicated as “Frequently” and “Always” used; ask further about ‘consent’]**?**

**3.** [Follow-up to Q153**] Please elaborate why, in your view, during the pandemic**

- **you changed the legal basis/condition that you would normally rely on** [when answer ‘No’ was chosen in the survey]**?**

**1.2 For participants who were directed to the pathway of questions that follows if answer “One legal basis” was chosen to Q150, and ‘Yes, it should be a combination’ to Q146 “Do you have a formal training in data protection?**

**1.** [Follow-up to Q154**] Please elaborate why you rely mostly on the following legal bases for the primary use of personal data for research and safety purposes** [cite the legal basis which they indicated as “Always” and “Most of the time” used; ask further about the use of ‘consent’ (e.g. if they chose ‘never’ - why never]**?**

**2.** [Follow-up to Q155**] Please elaborate why you rely mostly on the following special conditions for the primary use of personal data for research and safety purposes** [cite conditions which they indicated as “Always” and “Most of the time” used]**?**

**3.** [Follow-up to Q156**] Please elaborate why, in your view, during the pandemic**

- **you changed the legal basis/condition they would normally rely on** [when answer "No” was chosen in the survey]**?**

TOPIC 2: SECONDARY USE

**2.1** [Follow-up to Q160, if answers “Sometime”, “About half the time”, “Most of the time”, “Always” are chosen] **For the secondary use of personal data for research, how do you apply the presumption of compatibility**?

**2.2** [Follow-up to Q160, if answer “Never” is chosen] **For the secondary use of personal data for research, please elaborate why you do not rely on the presumption of compatibility**?

**3.1** [Follow-up to Q162, if answers “Sometime”, “About half the time”, “Most of the time”, “Always” are chosen ]  **Why do you choose to conduct the compatibility assessment?**

**3.2** [Follow-up to Q162, if answer “Never” ] **Why do you not conduct the compatibility assessment?**

4. [Follow-up to Q163] **Please elaborate on the reasons whythe following elements** [to cite the participant’s answer in the survey] **of the compatibility assessment are the most important?**

5. **Do you think that the rules for the secondary use of personal data for scientific research are or should be applied differently when**:

- the original controller – meaning the controller who processed the data for primary use – re-uses it for further purposes
- another controller – meaning a controller to whom the personal data has been shared by the original controller – re-uses the data for further purposes?

Please elaborate (regardless of the answer: Yes/No)

**6. [Precision medicine question]** Precision medicine is a new therapeutic approach that promises to cure the incurable by choosing treatment based on the individual’s genetic profile. In practice, the precision medicine mechanism consists of a diagnostic device which is combined with a drug. The patient is tested so that his genetic makeup could be determined and that it could be measured whether he is likely to respond to the drug. There are new therapies, mainly in oncology at the moment, that only work on specific mutations.

**In your experience, are there any differences in complying with the principle of lawfulness – i.e. the choice of a legal basis for primary or secondary use – when the clinical study that is conducted by your organisation employs a precision medicine approach?**

- **If yes: please elaborate**
- **If no: please elaborate**

TOPIC 3: Transparency

1. When your organisation prospectively collects personal data for research purposes, how do they inform study participants about it?

2. How would you ideally provide information?

3. During the pandemic and lockdowns, was there a change in the way that your organisation provided information to study participants about the processing of their personal data?

4. How would you ideally provide information if there is another lockdown/a new pandemic?

5. In situation where your organisation re-uses personal data obtained from another data controller, do they inform the study participants about the re-use?

- If no: Why? *For instance, does your organisation rely on the exceptions in Article 14(5)(b) or Article 11 of the GDPR [the two provisions will be explained]?*
- If yes: How? *For instance, public announcement? Or do they inform the study participants with the assistance of the original data controller, based on contractual agreements?*

- if their organisation has never considered the assistance of the original data controller, explore why; would they; what do they think about the possibility?

6. [Precision medicine question] In your experience, are there any differences in complying with the principle of transparency when the clinical study that is conducted by your organisation employs a precision medicine approach?

- If yes: please elaborate
- If no: please elaborate

TOPIC 4: Data control

*It is through the data protection legal framework that individuals are empowered and receive control over their personal data (Recital 7 GDPR).*

1. What do you think about data control of individuals over their personal data in clinical research?

2. How do you think data control by individuals/participants could work in practice?

3. There is a new legislation on data sharing at the European level (called the Data Governance Act), wherein the concept ‘data altruism’ is proposed. This refers amongst others to ‘the consent by data subjects to process their personal data, for purposes of general interest, such as scientific research purposes or improving public services’. So persons giving consent to process their data for purposes of public interest. What is your view of newly proposed data altruism mechanism?

TOPIC 5: Communication with ethics committees

1. [Follow-up to Q174 and Q175]

- [if answer “Yes” to either Q] *For instance, does your organisation follow their advice? What happens if the organisation does not follow the advice?*
- [If answer "No” to either Q] **Please share your thoughts: would you prefer if ethics committees would advise on the use of a specific legal basis? Why**?

2. [Follow-up to Q176]

- [if answer “Yes”] **Please share your thoughts about situations where ethics committees would insist on the use of specific techniques for pseudo- or anonymisation?**
- Possible follow-up questions: *For instance, does your organisation follow their advice? What happens if the organisation does not follow the advice?*
- [If answer “No”] **Please share your thoughts: would you prefer if ethics committees would advise on the use of specific techniques for pseudo- or anonymization? Why?**

3. [Precision medicine question] In your experience, are there any differences in the advice provided by the ethics committee on data protection issues, when the clinical study that is conducted by your organisation employs a precision medicine approach?

TOPIC 6: Challenges prior to and during the pandemic

1. [Follow-up to Q178] **In the survey, you indicated the following topics** [to insert based on the participant’s answer] **as the ones with which you and/or your organisation** **has experienced the most challenges prior to the pandemic. Could you please share concrete examples related to the topics?**

2. [Follow-up to Q178] Could you **please elaborate why these topics are the most challenging**, according to you?

3. [Follow-up to Q179] **In the survey, you indicated that the following topics** [to insert based on the participant’s answer] **as the ones with which you and/or organisation experienced the most challenges during the pandemic. Could you please share concrete examples related to the topics?**

4. [Follow-up to both Q178 and Q179]

- in case the challenges are the same: **Why, according to you?**
- in case the challenges are different: **Why, according to you?**

5. [Follow-up to Q179] **Could you please elaborate why these topics are the most challenging, according to you?**

6. [Follow-up to Q179] **What, do you think, is the cause of these challenges?**

7. [Follow-up to Q179] **How would you normally address them?**

8. [Follow-up to Q178 and 179]

[Follow-up to Q49 and 50] **How would you ideally address challenges experienced prior to and during the pandemic or ask to be addressed at EU/national level?**

9. [Precision medicine question] In your experience, are the challenges different, when the clinical study that is conducted by your organisation employs a precision medicine approach?

Closing questions

1. As a final question, do you have any further comments or issues which you think may be relevant for the compliance with the GDPR for clinical studies? If yes, which one(s)?

2. Do you have any questions for me?

3. EC members

TOPIC 1: Lawfulness

2. **How do you understand your role as an ethics committee in data protection matters**?

(*if they do not see a role for themselves at the moment:* Do you think that you should have a role as an ethics committee in data protection matters?)

3. [Follow-up to Q85]

- [if answer “Yes”] **Could you please give me an example of activities in the scope of which the ethics committee processes personal data?**
- [if answer “No”] **What do you think about having to review reports about, for instance, serious adverse events**?

4. [Follow-up to Q98]

- [if answer “Yes”] **Could you please share more about the type of situations in which you, as an ethics committee, chose to suggest a legal basis?**

5. [Follow-up to Q99 and 100] **Please elaborate why you mostly suggest the following legal bases/conditions** [cite the legal basis/condition which they indicated as “Sometimes”, “About half the time”, “Most of the time” and “Always” used]?

6. [Follow-up to Q102] **Please elaborate why during the pandemic, you advised researchers**

- - to rely on the same legal basis/conditions [when answer “Yes” was chosen in the survey]
  - to rely on different legal basis/conditions [when answer “No” was chosen in the survey]?

8. **[Precision medicine question]** Precision medicine is a new therapeutic approach that promises to cure the incurable by choosing treatment based on the individual’s genetic profile. In practice, the precision medicine mechanism consists of a diagnostic device which is combined with a drug. The patient is tested so that his genetic makeup could be determined and that it could be measured whether he is likely to respond to the drug. There are new therapies, mainly in oncology at the moment, that only work on specific mutations.

In your experience, are there OR should there be any differences in complying with the principle of lawfulness – i.e. the choice of a legal basis for primary or secondary use – when a clinical study employs a precision medicine approach?

- If yes: please elaborate
- If no: please elaborate

TOPIC 2: Transparency

1. In your opinion, what would be the ideal way to inform study participants about the use of their personal data for research purposes in the context of a study in which they participate?

2. In your view, which current rules (EU and/or national) prevent data controllers from informing study participants in the ideal way you suggested?

3. In your opinion, what would be the ideal way to keep study participants informed about the secondary use of their personal data (e.g. after the end of the clinical trial in which they have participated)? Please specify 1) when the new project is conducted by the original data controller, 2) when the new project is conducted by a new data controller who has not directly obtained the data from the patients.

4. In your view, which current rules (EU and/or national) prevent data controllers from informing study participants in the ideal way you suggested above?

TOPIC 3: Data control

*It is through the data protection legal framework that individuals are empowered and receive control over their personal data (Recital 7 GDPR).*

1. What do you think about data control of individuals over their personal data in clinical research?

2. How do you think data control by individuals/participants could work in practice?

3. There is a new legislation on data sharing at the European level (called the Data Governance Act), wherein the concept ‘data altruism’ is proposed. This refers amongst others to ‘the consent by data subjects to process their personal data, for purposes of general interest, such as scientific research purposes or improving public services’. So persons giving consent to process their data for purposes of public interest. What is your view of newly proposed data altruism mechanism?

TOPIC 4: Challenges prior to and during the pandemic

1. [Follow-up to Q107] In the survey, you indicated the following topics [to insert based on the participant’s answer] as the ones with which researchers have experienced the most challenges prior to the pandemic. Could you please share concrete examples related to the topics?

2. [Follow-up to Q107] Could you please elaborate why these topics are the most challenging, according to you?

3. [Follow-up to Q107] What, do you think, is the cause of these challenges?

4. [Follow-up to Q107] How do you think that they are normally addressed by researchers?

5. [Follow-up to Q107] How do you think they should be ideally addressed?

6. [Follow-up to Q108] In the survey, you indicated that the following topics [to insert based on the participant’s answer] as the ones with which researchers have experience the most challenges during the pandemic. Could you please share concrete examples related to the topics?

7. [Follow-up to Q108] Could you please elaborate why these topics are the most challenging, according to you?

8. [Follow-up to Q108] What, do you think, is the cause of these challenges?

9. [Follow-up to Q108] How do you think that they are normally addressed by researchers?

10. [Follow-up to Q108] How do you think they should be ideally addressed?

11. [Follow-up to both Q107 and Q107]

- in case the challenges are the same: Why, according to you?
- in case the challenges are different: Why, according to you?

12. [Precision medicine question] In your experience, are the challenges different, when a clinical study employs a precision medicine approach?

Closing questions

1. As a final question, do you have any further comments or issues which you think may be relevant for the compliance with the GDPR for clinical studies? If yes, which one(s)?

2. Do you have any questions for me?
