# Supplementary material for: Challenges related to data protection in clinical research before and during the COVID-19 pandemic: An exploratory study
Source: Front Med (Lausanne). 2022 Oct 10;9:995689. doi: 10.3389/fmed.2022.995689 (PMC9589288; doi:10.3389/fmed.2022.995689)
Supplement: Supplementary file 3 [file Data_Sheet_3.DOCX]

Supplementary Material 3

# Supplementary Figures

**Supplementary Figure 1: How often key research stakeholders rely on or advise about the use of one of the Article 6(1) GDPR legal bases: (A) DPOs/legal experts, (B) Investigators, (C) EC members**

**(A)**

**
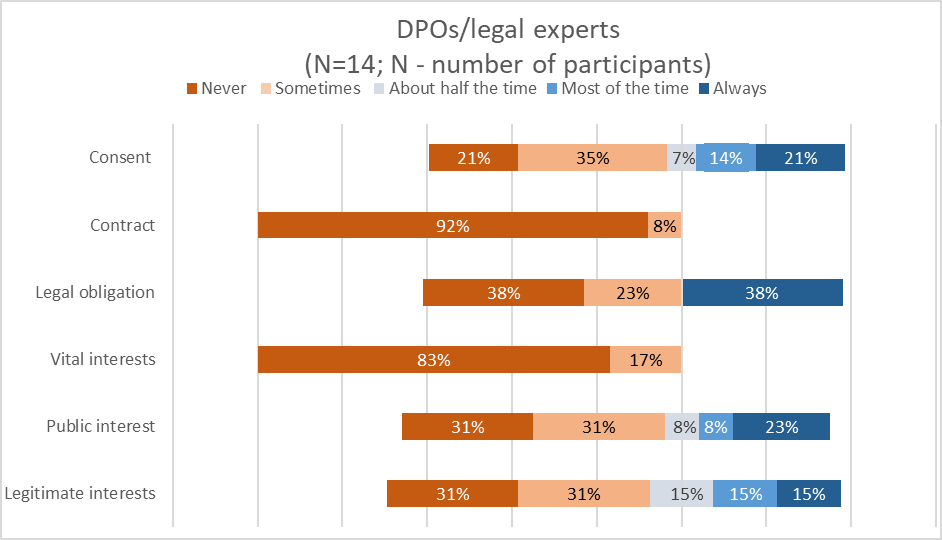
**

**(B)**

**
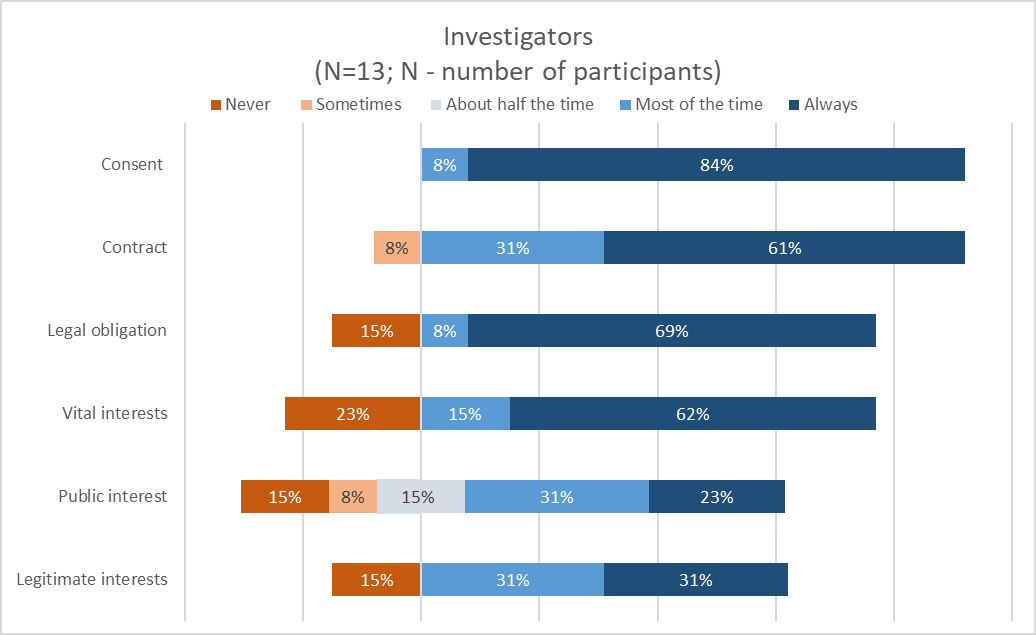
**

**(C)**

**
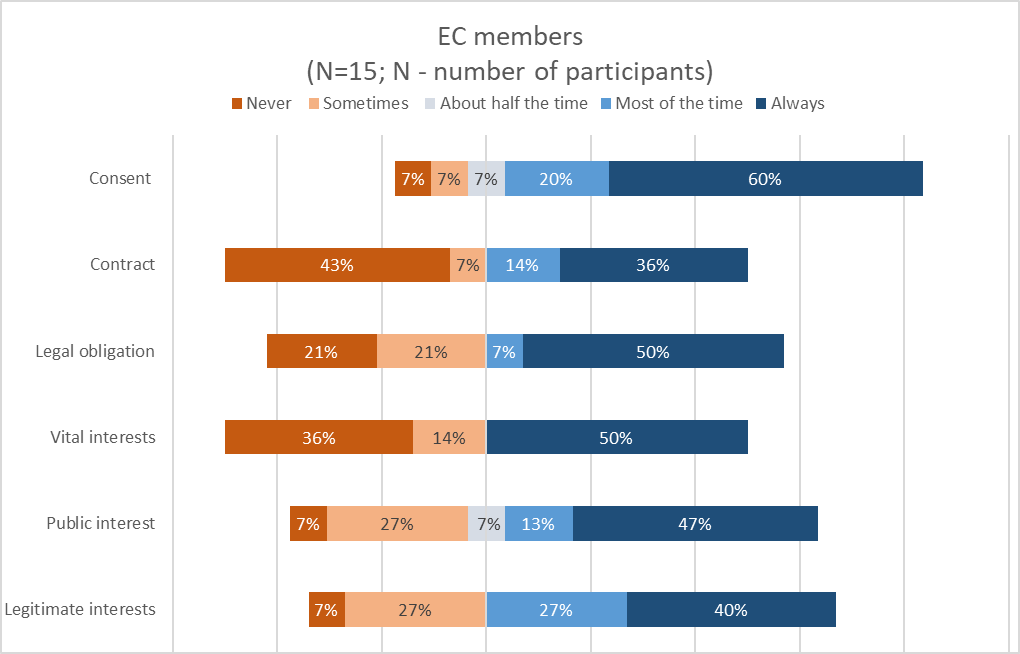
**

**Supplementary Figure 2: How often key research stakeholders rely on or advise about the use of one of the Article 9(2) GDPR special conditions: (A) DPOs/legal experts, (B) Investigators, (C) EC members**

**(A)**

**
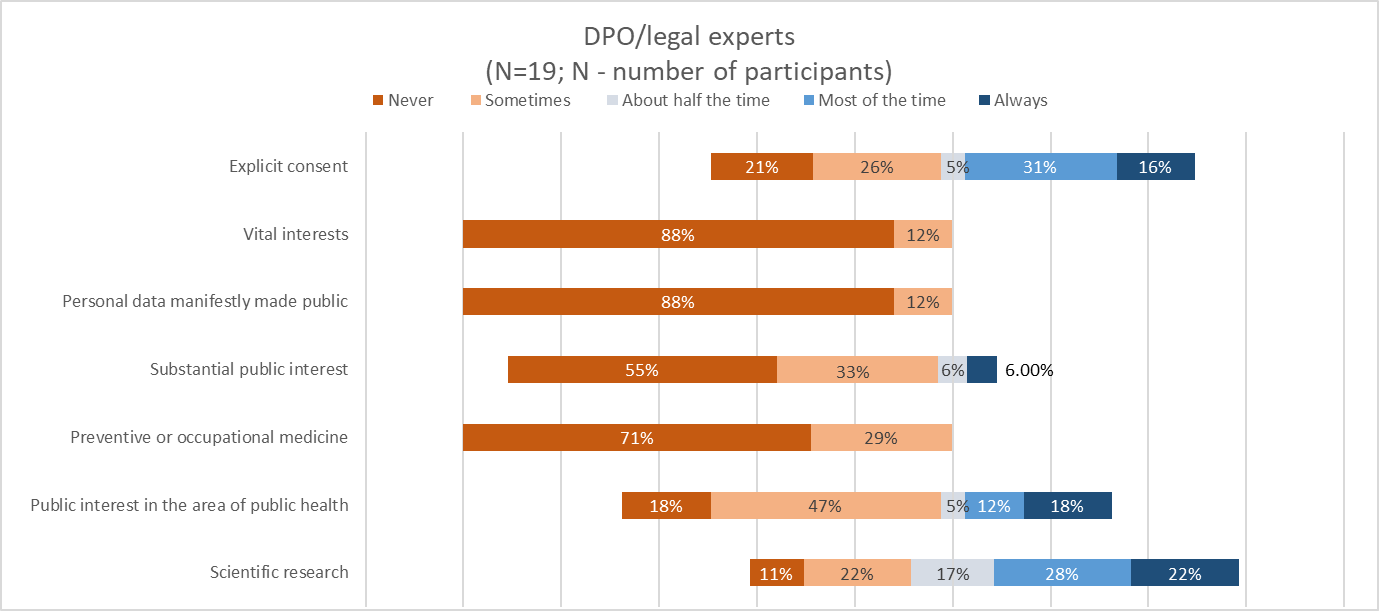
**

**(B)**

**
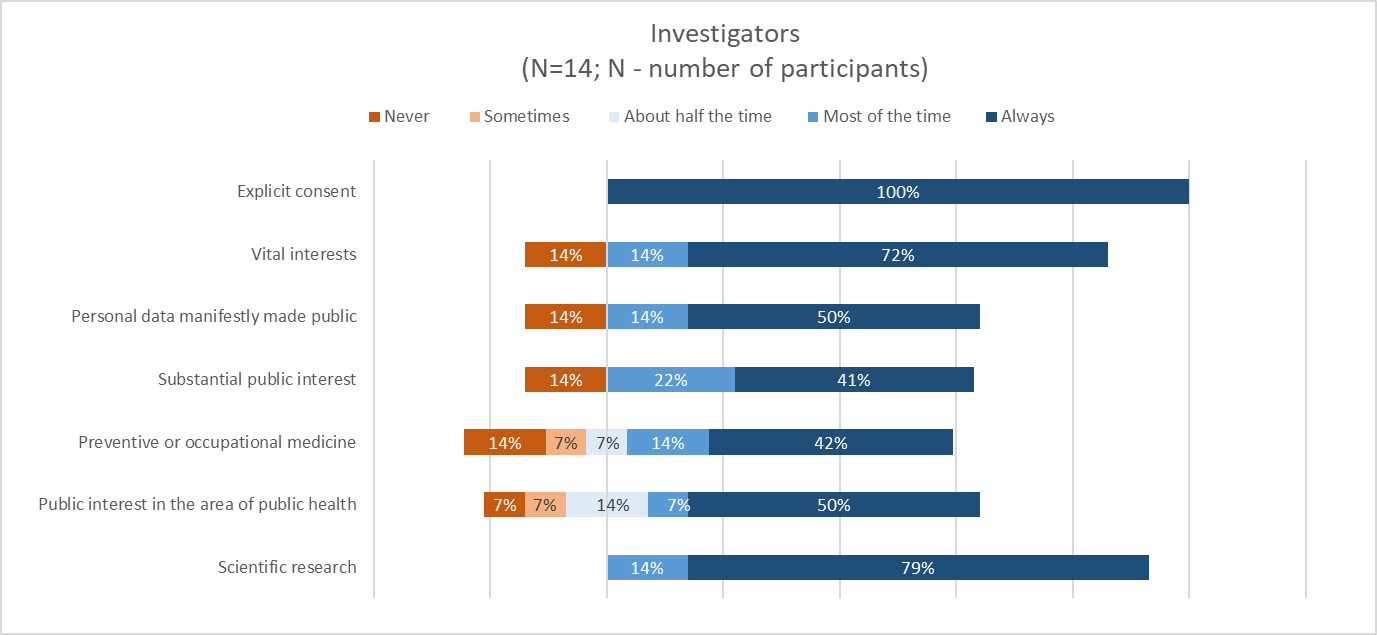
**
